# Feasibility and pharmacokinetic evaluation of a needle‐free injector for delivering high concentration antibody formulations

**DOI:** 10.1002/btm2.70063

**Published:** 2025-08-26

**Authors:** Alexander Josowitz, Arjun Sree Manoj, Danielle Laiacona, Marc Pelletier, Diana Molano, Samuel Jennings, Cassie Ng, Charlotte Antoni, Grace Chan, Robert Mahoney, Sanket Patel, Ellen‐Marie Koehler‐Stec, Marc Retter, Joel Kantrowitz, Bindhu Rayaprolu, Eric Holowka, Amardeep Singh Bhalla, Mohammed Shameem

**Affiliations:** ^1^ Regeneron Pharmaceuticals, Inc. Tarrytown New York USA; ^2^ Portal Instruments Cambridge Massachusetts USA

**Keywords:** high concentration antibodies, needle‐free, porcine pharmacokinetics, subcutaneous delivery

## Abstract

The increased preference amongst health care providers and patients for subcutaneous (SC) administration of biologics has necessitated the development of higher concentration formulations to maintain doses similar to intravenous (IV) products. These formulations possess manufacturing and administration challenges; particularly high concentration monoclonal antibody (mAb) formulations push the limit of injectability. Furthermore, patient‐centric considerations, such as pain and fear of needles (trypanophobia), can lead to compliance deviations for long‐term treatments. This study presents a set of evaluations of a novel computer‐controlled, needle‐free injector (NFI) design that can deliver 2.0 mL of a high viscosity (50 cP) mAb formulation into the SC space. Critical attributes such as antibody purity, aggregation, color, turbidity, and charge heterogeneity were evaluated before and after ejection and demonstrated minimal change compared to ejection from a 27‐gauge needle and syringe (N&S). Furthermore, the device functionality was evaluated in a novel ex vivo pig skin model, demonstrating the ability to accurately deposit a 2.0 mL dose at an appropriate depth in the SC tissue, though requiring 8% greater fill volume than an N&S. An in vivo Yorkshire pig model was used to understand the pharmacokinetic (PK) profile of the NFI in comparison to a N&S. Clearance (CL), the observed peak concentration in serum (*C*_max_), the time until *C*_max_ (*T*_max_), area under the concentration‐time curve extrapolated to infinity (AUC_inf_), and half‐life (*t*_1/2_) were all within 1.2 fold and considered similar between the NFI and N&S. A non‐significant difference in *T*_max_ was also observed. Bioavailability relative to IV administration was similar between the NFI (80.0%) and N&S (79.5%) groups. No concerning clinical observations and injection site reactions were observed. Ultimately, the NFI represents an advancement in SC delivery of high concentration mAb formulations with patient‐centric design. This device could facilitate clinical and at‐home use while complementing efforts to bridge IV and SC formulations.

**Translational Impact Statement**

This research report presents the evaluation of a novel NFI for the SC delivery of high concentration mAb formulations. This new, 2 mL design addresses some of the challenges patients and healthcare providers experience as industry has continued to shift toward SC delivery from IV. Furthermore, this evaluation utilizes ex vivo and in vivo porcine models to approximate the characteristics of human skin rather than small mammal models

## 1 INTRODUCTION

As the aging population increases, the prevalence of chronic and autoimmune diseases has increased.^1^, ^2^ As a result, the biologics market and associated investment has steadily been increasing, outpacing approvals of small‐molecule new molecular entities (NMEs) by the end of 2022.^3^ New biologics are generally large in molecular size (60 kDa–4 MDa), have trended toward higher concentrations, and are more complex in structure than small molecules. The diversity of biologic modalities allows for targeted treatments and therapies beyond the capabilities of small molecules alone, including monoclonal antibodies (mAbs), bispecific antibodies, antibody drug conjugates, nucleic acids, and gene therapies.^3^

Depending on the treatment schedule and condition, patients may take regular, intermittent, monthly, or annual injections. Irrespective of the treatment schedule, patients have reported struggling with adhering to a regiment due to pain and discomfort, the need for professional supervision or handling, the cost of travel to health care facilities, and prolonged injection times.^4^, ^5^, ^6^, ^7^, ^8^, ^9^ To improve the patient's experience, pharmaceutical companies are shifting drug formulations to more convenient self‐administered SC injection at home as opposed to in‐office IV administration. With the recent elevated health crises, such as COVID‐19, the management of illness away from the hospital and patient center has garnered more interest.^9^ Thus, many biologics are shifting to the SC route of administration.

Targeted therapies such as mAbs require larger volumes (>1.0 mL) to deliver the required dose at currently achievable concentrations.^9^ While there are drug delivery devices (such as needle‐based prefilled syringes and autoinjectors) that can perform large volume SC injections, injection times may range from 10 to 30s pressed against the skin, potentially leading to errors in administration such as lifting the device before the completion of the dose.^9^ They may also require larger needle gauges to compensate for the formulation properties such as the viscosity of the drug. This may lead to greater discomfort and anxiety toward performing the injection.^10^

Even with the added convenience and cost of at‐home self‐administration, drug delivery device design must account for how the patient performs these injections, changes in drug properties in different storage conditions, ease of following administration instructions, ease of use of the device, sharps disposal, and prevention of needlestick injuries.^6^, ^10^ From the patient‐centric perspective, the user may also experience needle phobia, anxiety, and stress from self‐administration, potentially leading to noncompliance and incorrect dosing. From the formulation perspective, dependent on the dosing frequency and dosing needed, these formulations may be highly concentrated, usually leading to more viscous solutions. These pose significant challenges for needle‐based syringes and autoinjectors, including needle clogging, high injection forces, and device failure, which could also impact patient adherence and HCP willingness to choose one treatment over another.

Needle‐free technology could address several of these challenges. Needle‐free technology can increase user freedom, avoid needlestick and needle infection/injuries, increase patient compliance, and be reusable (reducing biohazard risk and waste).^6^ It can also address the significant challenge of highly viscous formulations. There are several different types of NFIs, from spring systems to gas/combatcombustion powered devices.^6^

Historically, needle‐free systems were developed in the form of jet injectors for self‐administration of insulin and mass vaccination campaigns.^11^ One of the earliest needle‐free devices from the 1940s, the Hypospray, utilized a spring‐based propulsion system and was used for intradermal administration of insulin and intra‐articular administration of hydrocortisone to treat arthritis.^12^, ^13^ Many early needle‐free devices were multi‐use nozzle‐based technologies, while most modern iterations utilize a disposable cartridge system.^11^ The transition to disposable cartridge systems began in the 1990s, after the United States Food and Drug Administration (FDA) and military disavowed use of the previously widely adopted Ped‐O‐Jet and Med‐E‐Jet due to hepatitis B outbreaks related to improper decontamination practices of the nozzle between patients.^11^ Needle‐free vaccination has continued to be common for livestock and has been demonstrated to be more effective than traditional intramuscular injection in preventing disease transmission.^14^, ^15^ Newer devices with the disposable cartridge design have largely addressed these safety concerns, and many such devices have been marketed in the United States and internationally. Most prominently, the Biojector® 2000 (gas propelled) and Pharmajet Stratis® (spring powered) have emerged as prominent modern needle‐free technologies, though they have primarily been investigated for vaccination campaigns.^6^, ^16^ Additionally, newer designs for small volume NFIs have utilized lasers as the actuation mechanism for precise control of intradermal deposition.^17^ Advances in larger volume NF delivery have included novel nozzles, such as the multi‐orifice design by McKeage et al.^18^

More recently, needle‐free technology has been explored for hormone growth therapies, corneal drug application, neocollagenesis, intracavernosal therapies, and more.^6^, ^19^, ^20^, ^21^ However, these technologies also have limitations. Some devices have limited control over the pressure applied for the duration of the injection, may require additional manufacturing of reactants or primary containers, and may need periodic replacement of components (e.g. gas chambers for combustion propelled devices).^6^, ^7^

Portal Instruments® has previously developed the PRIME® needle‐free technology platform to address the significant challenges associated with current devices, including injection of viscous formulations; new, diverse modalities with distinct properties; a need for at‐home administration; needle‐based injuries; infections and waste; and current needle‐free technology limitations. The PRIME technology uses a computer‐controlled linear actuator to pressurize the fluid within a needle‐free cartridge and inject a highly collimated liquid stream through the skin. It also contains an internal feedback control system, enabling the injection depth to be tuned and allowing for precise delivery of a formulation. A closed‐loop system is created by sensing the plunger force and speed as the fluid is being delivered. The PRIME needle‐free technology platform has been tested clinically with a 0.68 mL SC administration configuration, demonstrating that injections of saline were safe, well‐tolerated, and strongly preferred to a 27‐gauge needle and syringe.^8^ The device has also been tested in ex vivo and in vivo swine studies with formulations over 120 cP.^10^

The PRIME NFI technology platform has also been tested clinically with the novel 2.0 mL SC format, confirming the feasibility and tolerability of injection of up to 2.0 mL saline into the human abdomen.^9^ However, the 2.0 mL SC NFI had not previously demonstrated injectate delivery of macromolecules into tissue and bioequivalence in animal models.

In this study, we present a set of in vitro and ex vivo feasibility, and in vivo PK evaluations of the 2.0 mL NFI to deliver 2.0 mL of a high viscosity (50 cP) mAb formulation into the SC space.

## 2 MATERIALS AND METHODS

### 2.1 Materials

The injection device used in this study was the proprietary Portal Instruments® 2.0 mL NFI. The NFI leverages the PRIME® needle‐free technology platform to deliver larger volumes (>1.0 mL) up to 2.0 mL. It consists of a computer‐controlled handheld injector and single‐use, needle‐free cartridge. The NFI uses a patented electromechanical actuator that drives the fluid through a small orifice (<200 μm in diameter) into a fine collimated jet stream that can penetrate the skin and travel to the intended tissue layer. Furthermore, the NFI software enables the injections to also be tailored to the properties of the drug, enabling the ability to precisely control the release of the fluid of a wide range of viscosities and densities. Similar to the PRIME platform, the NFI also has the same internal feedback control loop, modifying the injection velocity in real time.

Single‐use, needle‐free 2.0 mL cartridges were made of cyclic olefin polymer (COP) into a stainless‐steel cartridge sleeve of the NFI. The cartridge is manufactured with a novel nozzle design to collimate the fluid jet over a short length. To measure the force applied by the NFI to the skin, a skin contact force reader was constructed using load cells.

A 2.25 mL glass prefillable syringe with a 27‐gauge ultra‐thin wall needle and a 6 mM Spacer Device (8 mM needle, 2 mM spacer) (Regeneron Pharmaceuticals, Inc) was used to simulate a standard of care SC injection for the formulation used in the study. The spacer was 3D‐printed to adjust the needle insertion depth to target the SC area.

The test article used in this study was mAb A provided and formulated by Regeneron.

#### 2.1.1 Injector in vitro feasibility testing

mAb A with provided characteristics (Table 1) was filtered using a Millipore™ Stericup® PVDF vacuum filtration unit (0.22 μm) prior to filling by hand into the 2.0 mL COP cartridges in a laminar flow hood. The cartridges were stoppered using a custom stoppering tool (Portal Instruments®). Ten of each cartridge were reserved for Time = 0 (*T*0) analysis while 10 of each cartridge were reserved for each stability time point (described later). For injection feasibility analysis, 5 of the 2.0 mL cartridges were reserved to test with the NFI. Briefly, cartridges were loaded into the NFI and the mAb A material was injected into 50 mL Fisher Scientific Falcon™ tubes. Ejected material was pooled to provide enough for all analyses. Concurrently, formulation 1 (F1) was also filled into 2.25 mL syringes, stoppered with West Novapure® stoppers, and immediately ejected into 2R glass vials (Schott) as a direct comparator. Furthermore, 5 of the filled 2.0 mL cartridges were compressed with a plastic plunger to represent a “manual push” through the cartridge as a control for device operation stresses. Finally, a 2.0 mL pipette was used to transfer mAb A F1 from its bulk container into glass vials as a final control.

**TABLE 1:** mAb A properties.

Serotype	mAb concentration (mg/mL)	Formulation (F1)	pH	Density (g/mL)	Viscosity (cP)
IgG4	175	10 mM histidine, 5% sucrose, 0.15% polysorbate 80	6.0	1.069	54.3

Abbreviations: IgG4, immunoglobulin G4; mAb, Monoclonal antibody.

For the T0 material, and for all feasibility time points, the following analyses were performed: Visual inspection for visible particulates using an optical inspection box (Powerlook); color in comparison to BY reference solutions (Fluka); turbidity in comparison to reference suspensions (HACH StablCal); subvisible particulates though Micro‐Flow™ Imaging (MFI™ 5100, ProteinSimple); pH (Seven Excellence™ pH Meter, Mettler Toledo); concentration (SoloVPE® with Agilent Cary 60 Spectrophotometer, C Technologies Inc.); purity by size‐exclusion chromatography (Waters™ AQUITY™ H‐Class with Tunable Ultraviolet detector); and charge heterogeneity with imaged capillary isoelectric focusing (iCE3™ Charge Variant Analyzer, ProteinSimple).

Furthermore, for the initial F1 characterization, single point viscosity analysis was performed using the m‐VROC® manual viscometer at 25°C (Rheosense) and the density was measured using the DM5 density meter (Mettler Toledo).

### 2.2 Device compatibility testing

Based on the provided properties of mAb A (Table 1), the NFI was programmed with an injection profile to deliver 2.0 mL into the SC region. The injection profile consists of a biphasic profile: (1) Pierce phase, and (2) Fill phase. In the pierce phase, the fluid penetrates the skin to a desired tissue depth (e.g., SC space). In the Fill phase, the fluid jet velocity decreases, dispersing the fluid at the desired SC depth. The injection profile was then categorized by the average pierce force (APF).

To ensure the formulation and injection profile are compatible with the NFI at any storage temperature and stability timepoint, an ejection study was performed measuring the APF at different drug temperatures (room temperature at approximately 22°C and storage temperature of approximately 5°C) and storage stability timepoints (*T* = 0, 1, and 3 months). The mAb A formulation was ejected from the NFI onto a force test rig measuring the output jet stream force to record the injection profile and record the APF. For each test group at each storage condition, *N* = 5 ejections were performed.

#### 2.2.1 Formulation stability in cartridges

The COP cartridges were placed in temperature— and humidity‐controlled stability chambers lengthwise such that F1 would contact both the stopper and tip cap, considered a worse‐case condition for material contact. At each time point (Table 2), the cartridges were removed, brought to a sterile laminar flow hood, and contents sufficient for all analyses emptied and pooled into 2R glass vials. The same panel of assays described previously in the feasibility study was performed for each time point and storage condition, except for viscosity and density measurement.

**TABLE 2:** Cartridge stability study design.

Temperature/Humidity	Length of incubation
*T*0	*T*0
5°C	2 weeks, 1 month, 3 months
25°C/60% RH	2 weeks, 1 month, 2 months, 3 months
40°C/75% RH	1 week, 2 weeks, 3 weeks, 1 month

Abbreviations: RH, relative humidity, *T*0, time = 0.

#### 2.2.2 Ex vivo evaluation in Porcine skin

Ex vivo swine tissue was used as a model to characterize injection performance and demonstrate the NFI's ability to deliver the fluid to the target SC region. A total of *N* = 12 swine tissue samples were used. The tissue samples were harvested and sourced the day before testing from Premier BioSource with the properties shown in Table 3.

**TABLE 3:** Properties of ex vivo Swine tissue.

Species	*Sus scrofa domesticus*
Breed/Stock/Strain	Yorkshire
Sex	Female
Number	12
Source	Premier bioSource
Target weight range	50–60 kg
Injection sites	Lower abdominal area

The SC thickness between tissue samples was controlled to be similar for a more robust comparison between device performance. To prevent degradation during transit, tissue samples were shipped overnight in vacuum‐sealed bags stored on cold ice. To better simulate the tissue environment, the tissue surface temperature was heated to 34–37°C (source) and the tissue was stretched around a ball fixture to simulate tissue backpressure and intrabdominal pressure. For injection deposition testing in the ex‐vivo model, approximately 0.12% of Methylene blue or green food dye (Signature Select) was added to 60 mL of mAb A. Two different dyes were used to further differentiate injection boluses.

**FIGURE 1:**
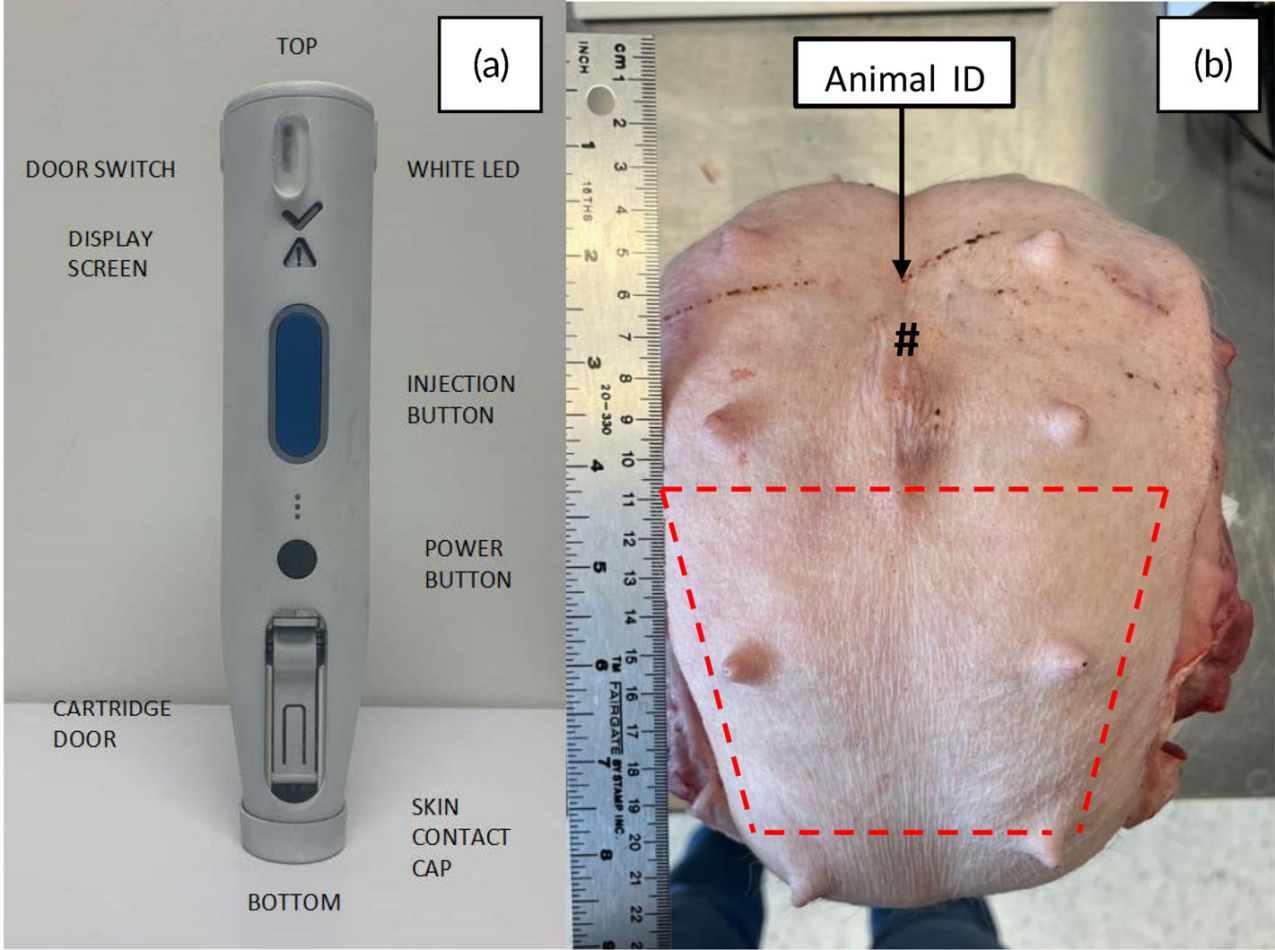
Novel NFI design denoting important user interface features (a) Representative ex vivo pig abdominal skin injection zone where a maximum of 8 injection*s* were performed (highlighted in red) with ruler for scale (b).

Depending on tissue and site viability, a maximum of eight injections were performed per tissue sample, four for each method of injection (i.e., N&S and NFI). The injections were performed in the denoted injection zone as seen in Figure 1. The specific sites of injections were defined pending injection site viability based on the physical boundaries of the mammary glands and the proximity of the harvest cut. The injections were performed with alternating injectate colors, for example, blue (Methylene blue) and green (food dye) to ensure bolus distinction.

A baseline (pre‐injection) photographic image of each injection site and a post‐injection photographic image of each injection site were taken. The post‐injection imaging occurred immediately after removing the NFI or N&S from the tissue's surface. These images were used to capture any injection site events that occur post‐injection, such as wheal (skin elevation) formation.

#### 2.2.3 Ex vivo delivered dose determination

Prefilled syringes and NFI cartridges with mAb A formulation were weighed before and after the injections were performed. Furthermore, Kimwipes were pre‐weighed and then used to absorb any fluid on the skin surface after injection for both NFI and comparator injections by gently placing on the injection site, without compressing the bolus. The Kimwipes were reweighed after fluid absorption and the difference was used to determine the weight of leaked or undelivered injectate. For the NFI, an additional Kimwipe was used to measure any fluid on the device that may have been ejected but did not penetrate the skin. These measurements were used to determine the appropriate cartridge fill volume to deliver the full intended dose into the tissue to inform the in vivo study design and quantify the fluid not delivered into the tissue, if any.

#### 2.2.4 Injection deposition characterization

**FIGURE 2:**
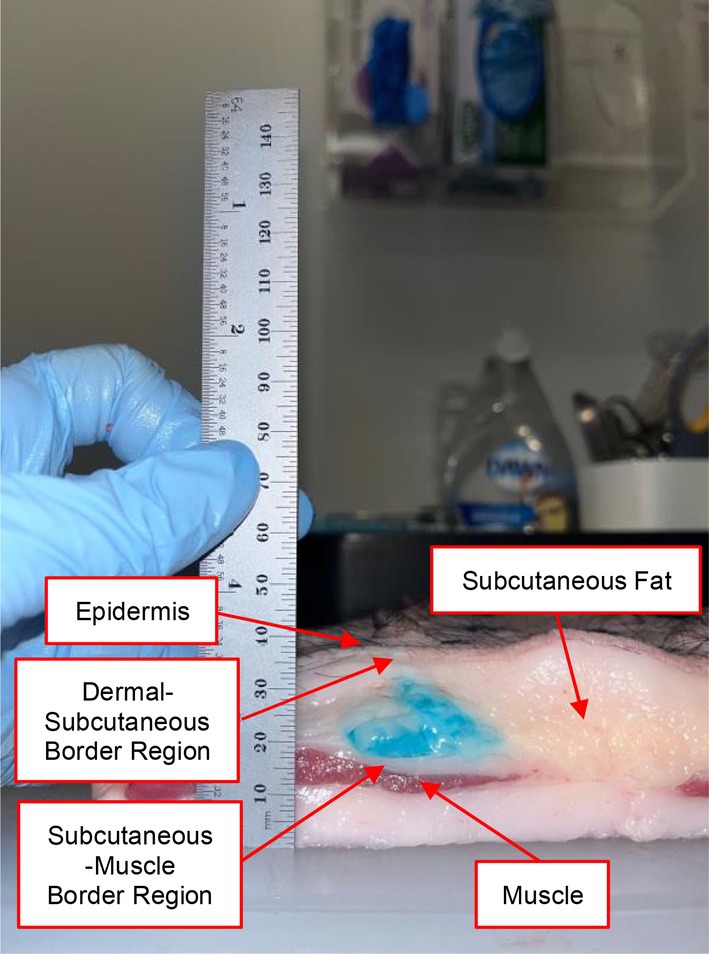
Representative cross‐sectional area of dissected swine abdominal tissue with an injection bolus visibly residing in the subcutaneous layer (blue). The prominent tissue layers are labeled and ruler provided for scale.

After all injections on a tissue sample were performed, the tissue was dissected across each point of injection transversely. Photographic images were taken to capture the cross‐sectional area with the injected boluses (as demonstrated in Figure 2). The cross‐sectional area was used to perform a qualitative analysis of each injection deposition.

For the qualitative analysis, each injection's performance was assessed and described according to the criteria outlined in Table 4 at the time of dissection. All observations were recorded.

**TABLE 4:** Injection deposition characterization categories.

Category 1	Category 2	Category 3	Category 4
Top of the bolus (blue dye) resides in the dermal‐subcutaneous border region OR subcutaneous region and bottom of the bolus resides in the subcutaneous‐muscle border region OR subcutaneous region.	Top of the bolus resides in the epidermis/dermis. Bottom of the bolus resides in the dermal‐subcutaneous border region.	Injectate pierces through the muscle and deposits below the muscle.	Injectate does not pierce the epidermis.
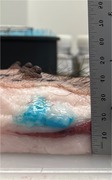	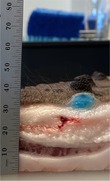	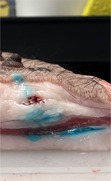	N/A

Abbreviation: N/A, not applicable.

#### 2.2.5 Animal welfare, husbandry, and ethics

All animal procedures were performed by the American Association for Accreditation of Laboratory Animal Care (AAALAC International) accredited personnel (CBSET) and in compliance with the United States Department of Agriculture (USDA), the Animal Welfare Act (AWA), and Animal Welfare Regulations (AWR). For this procedure (injection), the animals were housed prior to implantation for a minimum of 7 days. Animals were housed under conditions that met or exceeded requirements as set forth in the USDA AWA/AWR. Environmental conditions (temperature, relative humidity, and light) within the animal facility were monitored. Swine were offered Purina Lab Diet or Kent Nutrition diet once daily. Potable water was provided ad libitum to all animals.

#### 2.2.6 In vivo pharmacokinetic evaluation in Yorkshire pigs

Female Yorkshire pigs (*n* = 4), aged 4–6 months with weight between 49.0 and60.6 kg, were assigned to groups for testing based on weight. Animals underwent a single interventional procedure on Day 0 in which animals were injected with mAb A by SC injection with a pre‐filled N&S (Group 1); by SC injection with a prototype NFI (Group 2); or by IV injection (Group 3), as described in Table 5. Animal health, including treatment site observations, clinical observations, and body weights/body condition scores were generally monitored at pre‐determined, regular intervals. Blood samples for the PK analysis were collected at predetermined intervals. On 10 weeks ±2 days, animals were euthanized; no necropsy was performed.

**TABLE 5:** In vivo pharmacokinetic study design.

Group	Number of animals	Description of treatment	Injections per animal	Interim procedures	Study end point
1	4	Single 2 mL SC injection of test article via prefilled syringe with 27 Ga needle and spacer	1	Blood collection, Draize scoring, Clinical observation	10 weeks ± 2 days
2	4	Single 2 mL SC injection of test article via prototype needle‐free injector	1	Blood collection, Draize scoring, clinical observation	10 weeks ± 2 days
3	4	Single 2 mL IV injection of test article via prefilled syringe with standard needle	1	Blood collection, Draize scoring, clinical observation	10 weeks ± 2 days

Abbreviations: IV, intravenous; SC, subcutaneous.

Animals were not provided daily food ration prior to anesthesia for the injection procedures to avoid vomiting. Animals were administered either tiletamine‐zolazepam (intramuscular injection) or isofluorane (inhalation). Supportive IV fluids were administered through a peripheral vein catheter, and lubricant was applied to the eyes. Buprenorphine (0.01 mg/kg) was administered as pre‐emptive analgesia to animals in Group 2. Treatment sites were prepared aseptically, shaved free of hair, and wiped with 70% isopropyl alcohol prior to administration.

#### 2.2.7 Delivered dose determination for subcutaneous injections

Delivered doses were determined as described for the ex vivo tissue evaluation in Section 2.2.3 immediately after injection; while the animals were still anesthetized.

#### 2.2.8 Clinical observation and injection site monitoring

Digital images of the injection site were captured immediately after injection and after surface fluid was absorbed. Health checks were performed daily; clinical observation was performed both prior to treatment and at least once daily before the end of the study. Body weights were measured, and body conditions were determined before treatment and weekly thereafter. Injection sites for animals in Group 1 and 2 were evaluated with Draize Scoring (Table 6) prior to injection and at pre‐determined times until resolution of any observed skin effects. The irritation score is noted as the sum of the individual erythema and edema scores.

**TABLE 6:** Skin reaction scoring system.

Score	Erythema	Edema formation
0	No erythema	No edema
1	Very slight erythema (barely perceptible)	Very slight edema (barely perceptible)
2	Well‐defined erythema	Slight edema (edges of area well‐defined by definite raising)
3	Moderate to severe erythema	Moderate edema (raised approximately 1.0 mm)
4	Severe erythema (beet redness) to slight eschar formation (injuries in depth)	Severe edema (raised more than 1.0 mm beyond the area of exposure)

#### 2.2.9 Blood collection and preparation

Whole blood samples (3–5 mL) were collected at designated time points and transferred to a serum separator tube. Blood samples were allowed to sit at room temperature for 30 min prior to centrifugation at 2000 *g* for 15 min to produce serum. Serum aliquots were stored below −70°C during transport and prior to analysis.

#### 2.2.10 Bioanalytical ELISA assay development

An enzyme‐linked immunosorbent assay (ELISA) was developed to measure levels of human immunoglobulin G (IgG) antibodies (non‐specific) in pig serum. A microtiter plate coated with custom mouse anti‐human IgG (fragment crystallizable) Fc mAbs (Regeneron) was used for mAb A capture. mAb A was detected using a custom biotinylated mouse anti‐human Ig, kappa light chain specific (Regeneron), followed by NeutrAvidin conjugated with horseradish peroxidase (HRP) (Invitrogen). SuperSignal™ ELISA Pico Chemiluminescent Substrate (Thermo Fisher Scientific™) was used to quantify mAb A concentration based on signal intensity. mAb A reference standards and quality controls diluted in 2% pig serum were used to generate a calibration equation to describe the relationship between mAb A concentration and luminescence, measured in relative light units (RLU). Softmax® Pro GxP (6.5.1) was used to compute the curve parameters. mAb A concentrations were corrected for dilution to determine the relative concentrations in neat pig serum. Once established, the developed ELISA assay was used to compute the concentrations of mAb A in the serum samples collected from animals to which mAb A was administered.

#### 2.2.11 Pharmacokinetic analysis

Concentrations of total mAb A were analyzed by noncompartmental analysis (NCA) using Phoenix®, WinNonlin® (Version 8.3, Certara, L.P.) with IV bolus (for IV doses) or extravascular administration (for SC doses) routes selected. The target doses of the test article, as described in the study protocol, were used for NCA. The actual administered doses were within 10% of the target dose. A set of PK parameters was determined, such as *C*_max_, *T*_max_, area under the concentration‐time curve (AUC), *t*_1/2_, CL, and volume of distribution (*V*_ss_).

No anti‐drug antibody (ADA) analysis was performed by bioanalytical assay. The potential impact of an ADA response was assessed by visual inspection of the individual concentration‐time profiles. As there was no evidence of impact on the PK profiles, no study data was excluded from analysis.

## 3 RESULTS

### 3.1 Injector in vitro feasibility testing

The mAb A formulation demonstrated minimal to no change in a majority of critical quality attributes after ejection from the NFI in comparison to the T0‐tested material, pipette manipulation, ejection from N&S, and manual expulsion from a filled cartridge (Table 7). The ejected material visually appeared essentially free from visible particulates and maintained similar color and clarity and pH. Furthermore, the changes in protein content between each manipulation were within acceptable instrumental error, and there was no discernible change in protein purity, high molecular weight (HMW) aggregates (Figure S1), and charge heterogeneity. With regard to sub‐visible particles, aspect ratio and edge effect filters (MFI™ Image Analysis Software) were applied to each data set to eliminate the counting of bubbles or excess silicone oil. It was observed that the material ejected from the syringes demonstrated a noticeable increase in subvisible particles larger than 10 μm from the *T*0 material and the pipette manipulation group, likely due to interactions with silicone oil. There was also an increase in particulates larger than 10 μm from *T*0 for the material manually pushed from the cartridge (53 particles) and from the NFI (186 particles); however, this increase was less substantial than for the N&S (643 particles). All groups fell within the guidelines specified in USP<787> regarding sub‐visible particulate limits per container for small volume (<100 mL) therapeutic protein injections.

**TABLE 7:** Summary of mAb A quality attributes after ejection from NFI.

Attribute			mAb A formulation				
	Test method		*T*0 (before ejection)	Pipette	N&S	Manual push	NFI
Appearance and physical properties	Visible particles		0	0	0	0	0
	Color		Not > BY4	Not > BY4	Not > BY4	Not > BY4	Not > BY4
	Clarity		Not >18 NTU	Not >18 NTU	Not >18 NTU	Not >18 NTU	Not >18 NTU
	pH		5.94	6.04	6.04	6.05	6.05
Sub‐visible particles	MFI™		12/mL >10 μm 7/mL >25 μm	35/mL >10 μm 0/mL >25 μm	641/mL >10 μm 21/mL >25 μm	67/mL >10 μm 5/mL >25 μm	163/mL >10 μm 42/mL >25 μm
Content	SoloVPE®		176.0 mg/mL	179.3 mg/mL	180.1 mg/mL	169.8 mg/mL	177.5 mg/mL
Purity and impurities	Size‐exclusion chromatography (SEC)	Monomer	98.4%	98.2%	98.2%	98.2%	98.2%
		LMW	0.03%	0.1%	0.1%	0.0%	0.0%
		HMW	1.6%	1.8%	1.8%	1.8%	1.8%
Charge heterogeneity	Imaged capillary isoelectric focusing (iCIEF)	Acidic	30.0%	29.6%	30.2%	30.0%	30.6%
		Main	55.9%	56.9%	57.7%	58.0%	56.2%
		Basic	12.1%	10.5%	10.7%	10.5%	10.8%

Abbreviations: HMW, high molecular weight; iCIEF, imaged capillary electrophoresis‐based isoelectric focusing; LMW, low molecular weight; mAb, monoclonal antibody; MFI, Micro‐Flow™ Imaging; NFI, meedle‐free injector; Not > BY4, mot greater than Ph. Eur. Reference Standard BY4; Not >18 NTU, Not greater than 18 nephelometric turbidity units; Ph. Eur., European Pharmacopeia; SEC, Size‐exclusion chromatography; *T*0, time = 0; VPE, vaculoar processing enzyme.

### 3.2 Device compatibility testing

**FIGURE 3:**
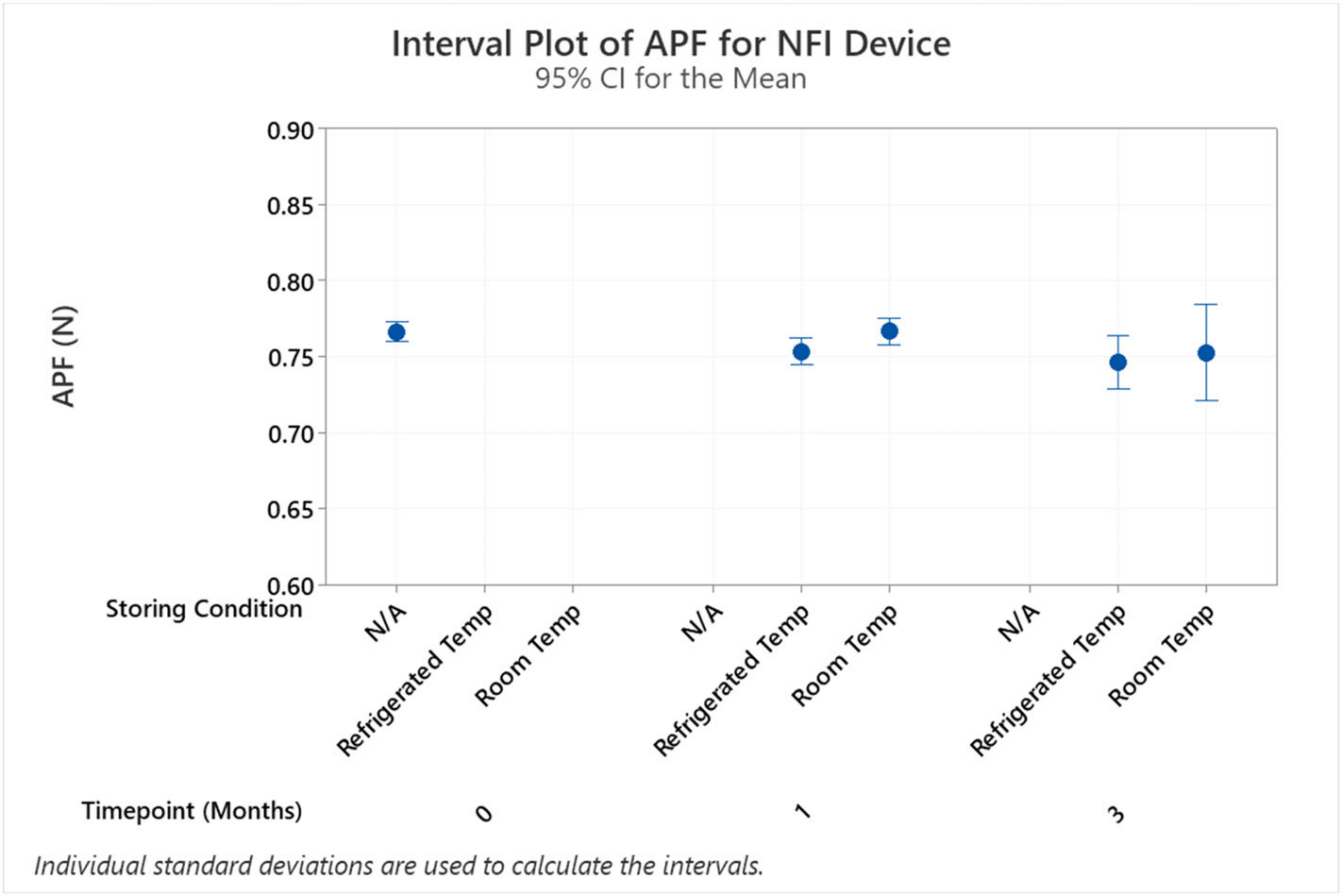
Average pierce force (APF) of mAb A formulation ejected from the NFI under multiple storage conditions. Storage of mAb A under refrigeration and room temperature conditions for up to 3 months did not significantly alter the APF. CI, confidence interval; mAb, monoclonal antibody; N/A, not applicable; NFI, needlefree injector.

For all ejections for each test group at each storage condition, the NFI completed the ejection successfully. No errors were present on the NFI interface. One‐way analysis of variance (ANOVA) test comparing APF values at each storage condition demonstrated no significant differences (*p* >0.05) (Figure 3).

The compatibility testing demonstrated that the intended injection profile was compatible with the formulation and NFI. At different storage temperatures and time points, the device performed similarly, showing robust ejection performance under different use cases.

### 3.3 Formulation stability in cartridges

**FIGURE 4:**
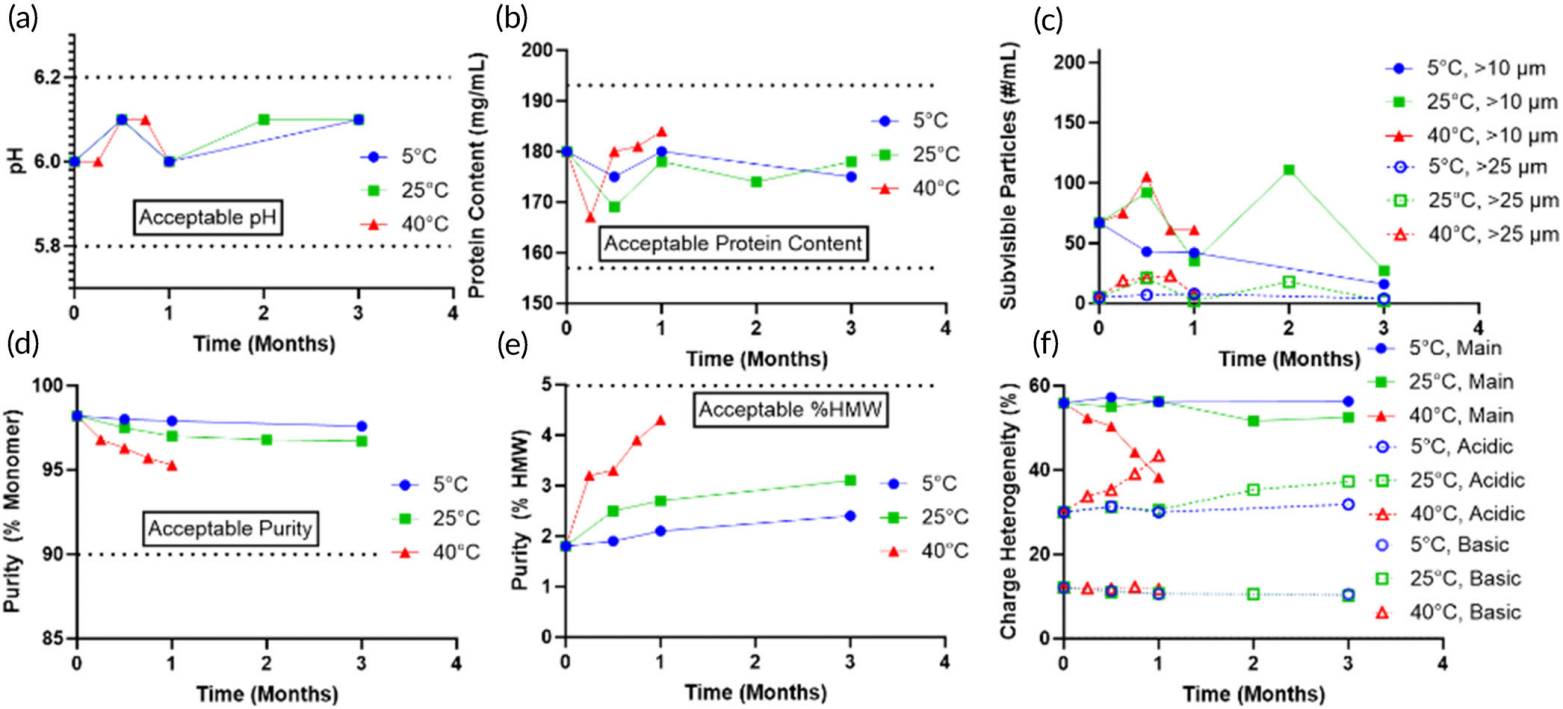
Summary of short term mAb A formulation stability in custom COP cartridges. Changes in pH (a) and concentration (b) were negligible for all storage conditions over the course of the study. No trend in sub‐visible particles greater than 10 and 25 μm was observed (c). Monomer purity decreased (d) and HMW species increased (e) in all storage conditions such that 5°C was more protective against aggregates compared to 25°C, which in turn was less destabilizing than 40°C storage. LMW change is not pictured as there was minimal detected at all times for all storage conditions. Charge heterogeneity remained stable at 5 and 25°C storage, though a shift toward more acidic species occurred at 40°C (f). COP, cyclic olefin polymer; HMW, high molecular weight; LMW, low molecular weight; mAb, monoclonal antibody.

With regard to stability within the custom COP cartridges, mAb A maintained quality attributes within pre‐established acceptance ranges throughout the duration of the study for up to 3 months in 5°C and 25°C/60% relative humidity (RH) conditions as well as the accelerated degradation conditions of 40°C/75% RH for 1 month. Visual analysis did not demonstrate any change from *T*0 in color, clarity, or visible particulates at any time points for each storage condition. Furthermore, pH (Figure 4a) and protein content (Figure 4b) demonstrated no trend over time, suggesting the stability of the formulation and no water evaporation from the cartridges. Subvisible particulates greater than 10 and 25 μm demonstrated no trend over time, nor among the different storage conditions, and remained similar to what was observed for the material that had been tested after expulsion from the NFI (Figure 4c). Over the time studied, there was a decreasing trend in purity (Figure 4d) and an increase in HMW species (Figure 4e) for all storage conditions, though the change was minimal for 5°C and more pronounced for 25°C/60% RH and 40°C/75% RH. The observed purity changes were within the pre‐established acceptance criteria for acceptable stability. At 5°C and 25°C/60% RH, there was minimal change in the distribution of charged species, while material stored at 40°C/75% demonstrated a shift toward more acidic species over time (Figure 4f).

### 3.4 Ex vivo evaluation in porcine skin

**FIGURE 5:**
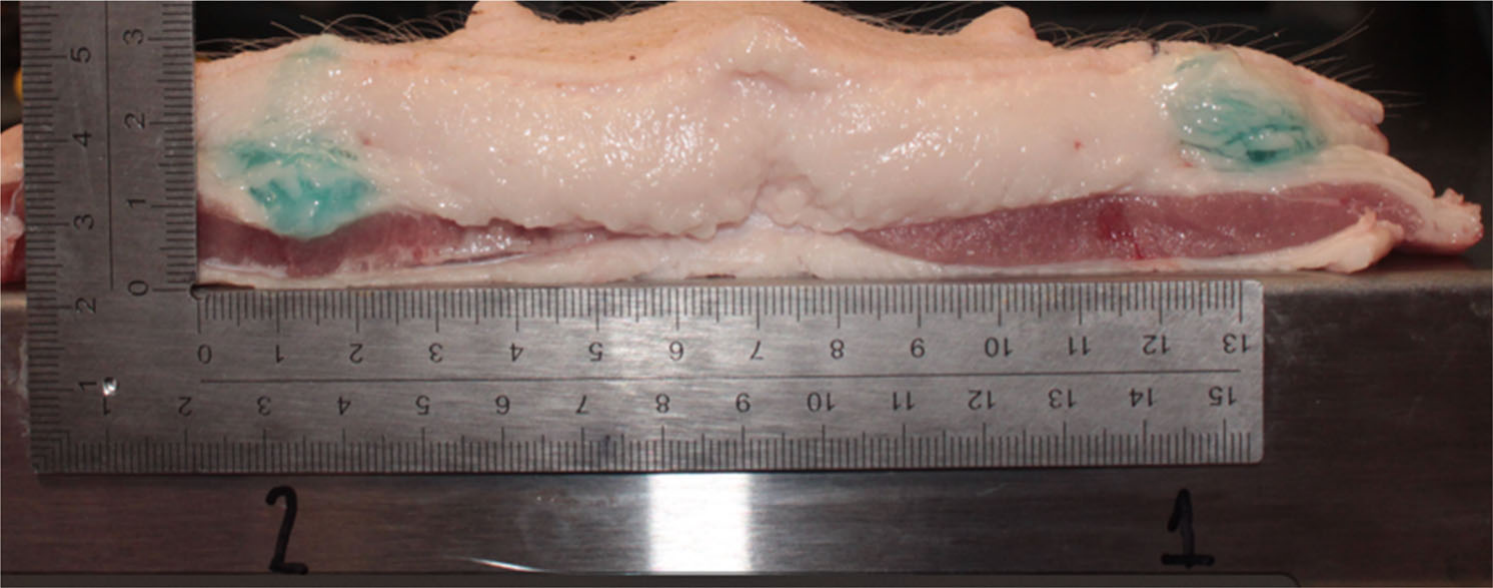
Representative image of ex vivo porcine skin after subcutaneous injections. Visible on the left (1) is an injection from the NFI, and on the right an injection from N&S (2). Ruler is provided for scale. N&S, needle and syringe; NFI, needlefree injector.

A total of 18 injections were performed with each device. With the NFI, 14 of 16 injections were deposited in the SC tissue layer (Category 1, Table 4), and 2 of 16 injections were deposited on top of the rectus abdominis muscle (Category 2, Table 4) (Figure 5). Similarly, with the N&S, 15 of 16 injections were deposited in the SC tissue layer (Category 1, Table 4), and 1 of 16 injections was deposited on top of the rectus abdominis muscle (Category 2, Table 4).

Moreover, both devices successfully injected into the tissue samples and delivered a mean of at least 2.00 mL of fluid. The N&S delivered an average of 2.20 mL, while the NFI delivered an average of 2.05 mL (Table 8).

**TABLE 8:** Dose delivery statistics for both NFI and N&S.

Delivery	*N*	Surface residual fluid (mL)	Undelivered fluid (mL)	Ejected volume (mL)	Delivered volume (mL)
		Mean ± Standard deviation	Mean ± Standard deviation	Mean ± Standard deviation	Mean ± Standard deviation
N&S	16	0.002 ± 0.003	0.002 ± 0.003	2.204 ± 0.025	2.202 ± 0.025
NFI	16	0.039 ± 0.024	0.079 ± 0.026	2.128 ± 0.006	2.049 ± 0.028

Abbreviations: N&S, needle and syringe; NFI, needle‐free injector.

Furthermore, the NFI took approximately 670 ms to inject the drug, while the N&S took approximately 12–15 s. In addition, the operator observation reported it required significant effort to press the plunger down for the N&S injection and keep it orthogonal to the skin surface.

### 3.5 In vivo evaluation clinical observations

**FIGURE 6:**
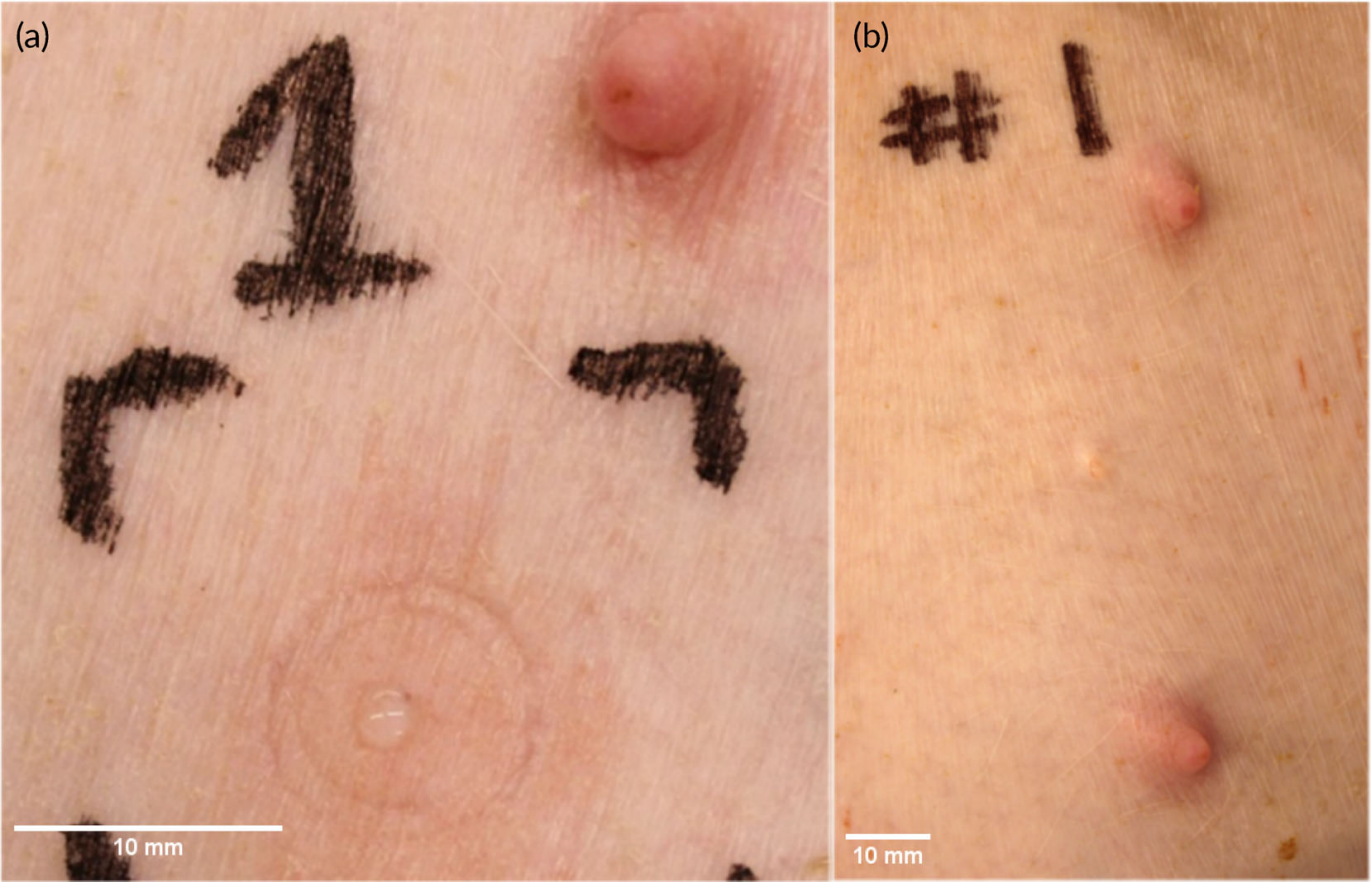
Representative images after in vivo subcutaneous injection with needle and syringe (a) and needle‐free injector (b).

All injections with the NFI and N&S were determined to have delivered at least 2.0 mL of mAb A formulation successfully following the dose determination procedure (Tables S1 and S2). Minor leakage was observed in both groups in some animals (Figure 6), and one animal in the NFI group demonstrated minor bleeding after injection. No clinical abnormalities were observed for any of the animals post‐injection nor throughout the course of the blood collection period. All animals gained weight and maintained good body condition throughout the course of the study (Tables S3–S5). Draize irritation scores for animals injected with the NFI and N&S were between 0 and 2 indicating mild erythema and/or edema, and signs of irritation for both groups tended to resolve within 3 h of injection (Tables S6–S13).

### 3.6 Pharmacokinetic bioanalysis

**FIGURE 7:**
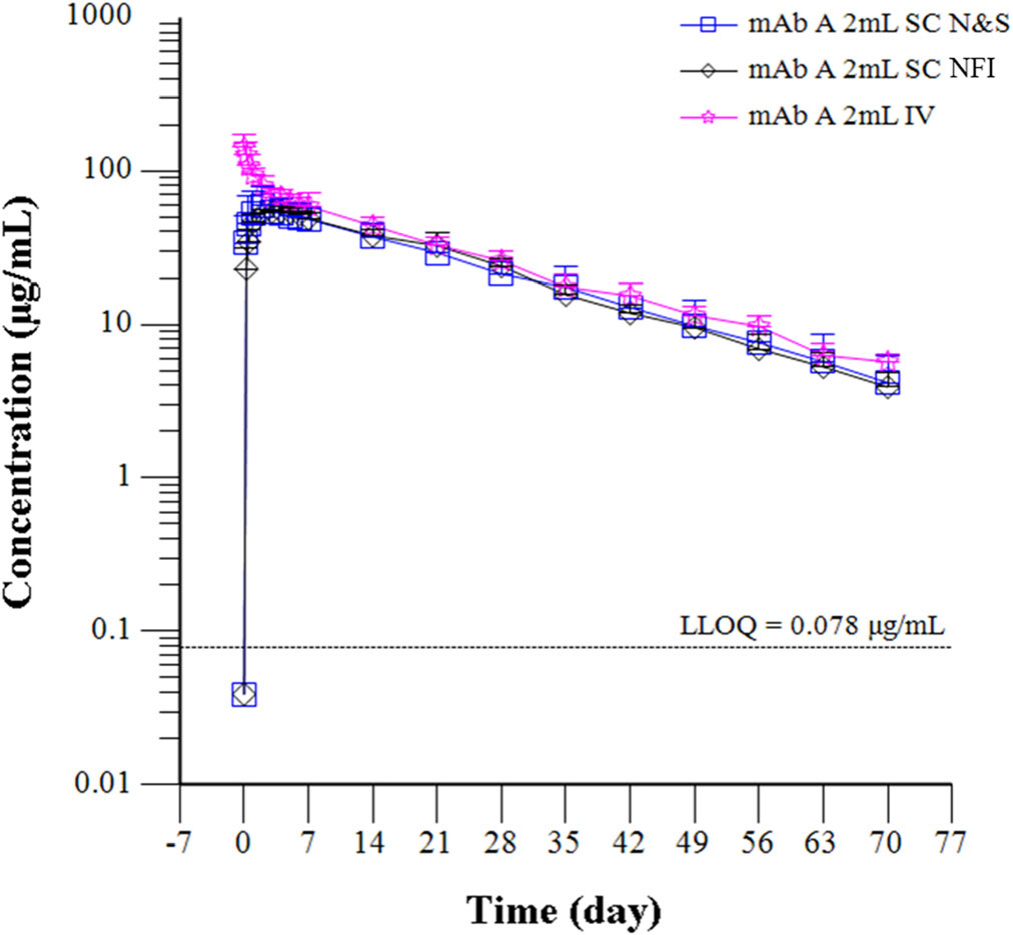
Mean total concentration of mAb A versus time. Profiles following single dose administration by needle and syringe (blue), NFI (black) and intravenous bolus (pink). Error bars represent standard deviation for each time point (*n* = 4). LLOQ, Lower limit of quantitation; mAb, monoclonal antibody; NFI, needle‐free injector; NFI, prototype needle‐free injector.

The lower limit of quantitation (LLOQ) for mAb A in neat serum was determined to be 0.078 μg/mL. Concentrations below the LLOQ were imputed as LLOQ/2 or 0.039 μg/mL for purposes of quantitation and representation (Table S14). Following the single dose of mAb A via N&S, NFI, or IV injection, the concentration‐time profiles of total mAb A were characterized by a short absorption phase (SC and SC NFI) or distribution phase (IV), followed by a single elimination phase through Day 71 (Figure 7). Concentration‐time profiles for individual animals by group are presented in Figures S2–S4.

Mean time to *T*_max_ was observed at approximately 2, 4 days, and 15 min for the SC, SC NFI, and IV dose groups, respectively. Mean *C*_max_ for the SC, SC NFI, and IV dose groups was 62.3, 56.1, and 142 μg/mL, respectively (Table 9). Mean *t*_½_ was 16.1, 17.9, and 18.9 days for the SC, SC NFI, and IV dose groups, respectively. Though a minor difference was observed in *T*_max_ between the SC and SC NFI groups, the *C*_max_ and *t*_½_ values were within 1.2‐fold and thus were comparable. Pharmacokinetic parameters and blood concentrations of mAb A for individual animals are presented in Tables S15–20.

**TABLE 9:** Mean pharmacokinetic parameters of total mAb A in serum following single dose.

Parameter	Unit	mAb A 2 mL SC				mAb A 2 mL SC (NFI)				mAb A 2 mL IV			
		*N*	Mean	SD	CV%	*N*	Mean	SD	CV%	*N*	Mean	SD	CV%
*C*_max_	μg/mL	4	62.3	20.2	32.4	4	56.1	3.03	5.41	4	142	30.4	21.4
*T*_max_	day or hour	4	1.63	0.750	46.2	4	4.00	0.816	20.4	4	0.250	0.00	0.00
AUC_inf_	day (μg/mL)	4	1630	520	32.0	4	1640	108	6.62	4	2050	233	11.4
*t*_1/2_	day	4	16.1	2.50	15.5	4	17.9	1.49	8.30	4	18.9	1.52	8.04
CL/F or CL	mL/day	4	224	99.2	44.4	4	199	13.0	6.54	4	160	17.6	11.0
*V*_ss_	mL	0	NC	NC	NC	0	NC	NC	NC	4	4150	410	9.90
Bioavailability	%	79.5				80.0				NC			

*Note*: *T*_max_ is presented in hours for the IV group and in days for the SC groups.

Abbreviations: AUC, area under the concentration‐time curve; AUC_inf_, AUC from time zero extrapolated to infinity; Bioavailability, (SC AUC_inf_/IV AUC_inf_) × 100; *C*_max_, peak concentration; CL, clearance for IV dosing; CL/F, apparent clearance for SC dosing; CV, coefficient of variation; IV, intravenous; mAb, monoclonal antibody; *N*, number of animals; NC, not calculated; NFI, needle‐free injector; SC, subcutaneous; SD, standard deviation; *t*_1/2_, half‐life; *T*_max_, time to *C*_max_; *V*_ss_, volume of distribution at steady state.

The observed mean AUC_inf_ of the SC, SC NFI, and IV groups was 1630, 1640, and 2050 day (μg/mL), respectively. The AUC_inf_ of the SC and SC NFI groups was within 1.1‐fold. Bioavailability (calculated using the ratio of the mean AUC_inf_ of the SC groups to that of the mean AUC_inf_ of the IV dose group) was comparable (79.5% and 80.0%) for the SC and SC NFI groups, respectively. Notably, the standard deviations of the *C*_max_, AUC_inf_, and CL/F for the N&S group were greater than those of the NFI group, indicating greater variability.

## 4 DISCUSSION

Initial in vitro testing demonstrated that the NFI was able to deliver a high concentration, high viscosity (54.3 cP) antibody formulation with minimal change to critical quality attributes. This represents a proof of concept of the feasibility of using needle‐free technology to administer mAbs. In particular, the NFI effectively delivered a formulation with viscosity higher than most commercial products, suggesting the ability to deliver beyond the limit of injectability for syringes and many autoinjectors. Formulation stability was acceptable within the customized COP cartridges; however, it was only performed for a short period for this initial evaluation. Longer stability studies must be performed to fully understand the cartridge shelf life, which is likely to be different depending on the formulation and specific antibody. One important limitation to the in vitro ejections was the means of collection into a plastic tube, which does not represent the physical properties of skin and allowed for greater air interface than was present for the ex vivo and in vivo injections. It is possible that the material experienced additional stresses due to the air interface and impact in the collection vessel that would be minimized during injection into tissue.

Both the NFI and N&S performed similarly in injection deposition and delivered at least 2.00 mL of mAb A formulation into the ex‐vivo tissue sample. However, the NFI delivered the material with a faster injection time (670 ms vs. 12–15 s). The operator of the study highlighted the user force input for the N&S was considerable, as expected for a 50 cP fluid. The study confirmed the injection profile and appropriate SC delivery for the NFI for the in vivo evaluation of the compound.

For the in vivo evaluation, to ensure a similar volume of fluid is injected into the animals, the N&S syringe volume was decreased from 2.20 to 2.04 mL, and the NFI container volume was kept at 2.20 mL due to fluid loss identified in the study. As a result, slightly more fluid appears to be required with the NFI to deliver a similar dose (approximately an 8% increase in fill volume).

With regard to the in vivo portion of this study, no clinical or injection site concerns were observed post injection with the NFI when compared to N&S. This suggests that the NFI could be similarly tolerable for human patients, as indicated by previous clinical studies.^8^, ^9^ Similar volume was delivered between the NFI and N&S (2.035 mL and 2.036 respectively). mAb A demonstrated comparable PK parameters following SC dosing using N&S and NFI, as exposure values (*C*_max_ and AUC_inf_) were within 1.1‐fold between the 2 SC dose groups; though the N&S demonstrated greater variability than the NFI group. The increased variability of the N&S group was due to one animal that had lower detected serum mAb A concentrations relative to the other animals in the group, despite the full dose having been successfully delivered, which might be attributable to inter‐animal variability. mAb A was detected in swine serum for all sampling time points, and no animals demonstrated evidence of ADA‐impacted concentration‐time profiles. Alternately, the *T*_max_ was greater for the NFI group compared to N&S though in the context of the variability of the N&S group and the similarities of the other PK parameters, this was not considered to represent a major difference in function between the two delivery methods. Furthermore, the similar bioavailability between both SC suggests that mAb A absorption was not altered through the use of the NFI. These results suggested no crucial alteration of mAb A's structure occurred during the injection process to affect tissue binding, absorption, or immunogenicity.

## 5 CONCLUSIONS

The NFI demonstrated comparability to the N&S in all aspects studied. The NFI maintained the integrity of mAb A after ejection and was able to deliver a high concentration, high viscosity formulation in an ex vivo pig skin model, as well as in live Yorkshire pigs. No concerning clinical observations were observed after injection with the NFI; PK profiles and parameters were comparable to those obtained with N&S.

Only a few differences were observed between the NFI and N&S. The NFI required a slightly larger fill volume to deliver a similar volume into the animal; though this was not considered to be a significant limitation. Alternatively, the N&S took longer to inject the complete dose and required significant effort to compress the plunger and keep the device steady.

Ultimately, the NFI represents an advancement in the ability to deliver high concentration and high viscosity mAb formulations through SC injection while preserving the physicochemical properties of mAb formulations, tolerability of standard injections, and PK. The NFI presents a platform for SC delivery of mAbs and possibly other large molecules while addressing many patient‐centric administration challenges, such as pain, fear of needles, and danger of needle stick injury.

## AUTHOR CONTRIBUTION

**Alexander Josowitz**: Conceptualization; methodology; investigation; writing—original draft; visualization; project administration and resources. **Arjun Sree Manoj**: Investigation; formal analysis; methodology; resources; project administration; visualization and writing—original draft. **Danielle Laiacona**: Methodology; conceptualization; resources; funding acquisition; project administration; validation; writing—review and editing. **Marc Pelletier**: Conceptualization; formal analysis; investigation; methodology; project administration; software; supervision; visualization; writing—review and editing. **Diana Molano**: Investigation; writing—original draft; visualization; formal analysis. **Samuel Jennings**: Formal analysis; investigation and visualization. **Cassie Ng**: Visualization; investigation and formal analysis. **Charlotte Antoni**: Investigation and visualization. **Grace Chan**: Formal analysis. **Robert Mahoney**: Investigation. **Sanket Patel**: Methodology; formal analysis and writing—original draft. **Ellen‐Marie Koehler‐Stec**: Supervision; validation and resources. **Marc Retter**: Validation; supervision and resources. **Joel Kantrowitz**: Writing—review and editing; validation and supervision. **Bindhu Rayaprolu**: Conceptualization; supervision; validation; writing—review and editing. **Eric Holowka**: Conceptualization; supervision; validation; funding acquisition; writing—review and editing. **Amardeep Singh Bhalla**: Conceptualization; supervision; validation; writing—review and editing. **Mohammed Shameem**: Supervision; resources and funding acquisition.

## CONFLICT OF INTEREST STATEMENT

AJ, DL, DM, GC, RM, SP, EMKS, MR, JK, BR, EH, ASB, and MS were employees and minor shareholders of Regeneron Pharmaceuticals, Inc. at the time this work was performed. ASM, MP, SJ, and CN were employees of Portal Instruments at the time this work was performed.

## Supporting information


**Data S1:** Supporting information

## Data Availability

The data supporting this study are available on request from corresponding authors. The data are not publicly available due to privacy or ethical considerations.
