# Supplementary material for: Feasibility and pharmacokinetic evaluation of a needle‐free injector for delivering high concentration antibody formulations
Source: Bioeng Transl Med. 2025 Aug 26;11(3):e70063. doi: 10.1002/btm2.70063 (PMC13247403; doi:10.1002/btm2.70063)
Supplement: Supplementary file 1 — Data S1: Supporting information [file BTM2-11-e70063-s001.docx]

1. Supplementary Information


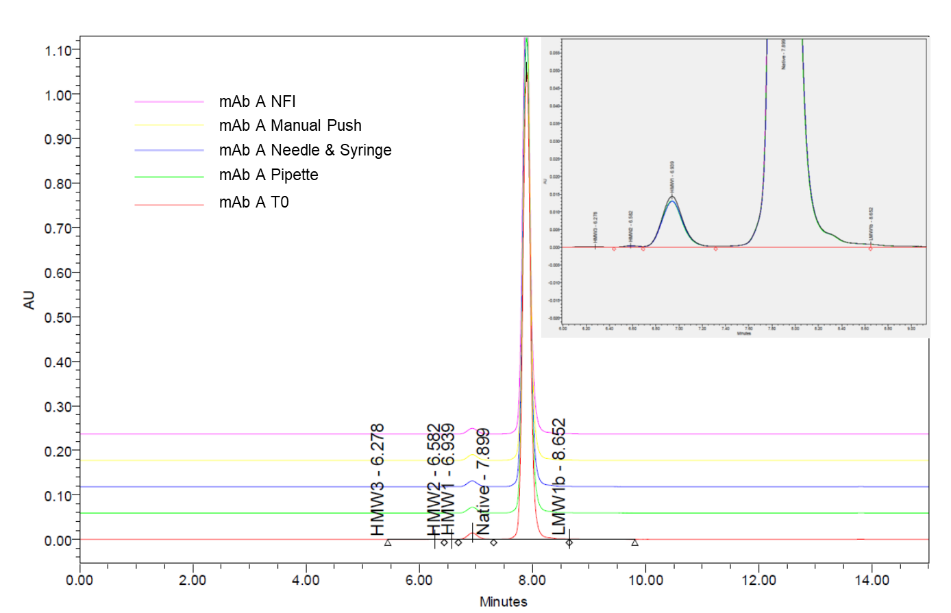


**Figure S1: SE-UPLC traces comparing protein purity after mAb A ejection from the NFI to other forms of manipulation. The insert shows a closer view demonstrating little change in HMW and LMW species.**

HMW, High molecular weight; LMW, Low molecular weight; mAb, Monoclonal antibody; NFI, Needle-free injector; SE-UPLC, Size-exclusion ultra performance liquid chromatography; T0, Time = 0

**Table S1: Summary of Delivered Dose Determination Administration with Subcutaneous Needle and Syringe**

| Group | | 1 | | | |
| --- | --- | --- | --- | --- | --- |
| Animal Number | | 1 | 2 | 3 | 4 |
| Device Name | | 2.25 mL Syringe and Needle with Spacer | | | |
| Lot Number | | Pilot002 Run 4 18-0037Z | | | |
| Route of Administration | | Subcutaneous | | | |
| Location of Treatment | | Right Lower Abdomen | Left Lower Abdomen | Right Lower Abdomen | Left Lower Abdomen |
| Pre- Injection | Weight of N&S without Spacer (g) | 8.3492 | 8.3394 | 8.3752 | 8.3611 |
|  | Weight of Kimwipe "A" (g) | 0.4560 | 0.4527 | 0.4594 | 0.4495 |
|  | Weight of Kimwipe "B" (g) | 0.4569 | 0.4534 | 0.4583 | 0.4614 |
| Post- Injection | Weight of N&S without Spacer (g) | 6.1836 | 6.1830 | 6.1961 | 6.1985 |
|  | Weight of Kimwipe "A" (g) | 0.4604 | 0.4663 | 0.4673 | 0.4535 |
|  | Weight of Kimwipe "B" (g) | 0.4569 | 0.4704 | 0.4559 | 0.4610 |
| Weight of Fluid Delivered with N&S without Spacer (g) | | 2.1656 | 2.1564 | 2.1791 | 2.1626 |
| Weight of Fluid Absorbed in Kimwipe "A" (g) | | 0.0044 | 0.0136 | 0.0079 | 0.0040 |
| Weight of Fluid Absorbed in Kimwipe "B" (g) | | 0.0000 | 0.0170 | -0.0024 | -0.0004 |
| Total Weight of Undelivered Fluid (g) | | 0.0044 | 0.0306 | 0.0055 | 0.0036 |
| Procedural Comments | | None | None | None | None |

N&S, Needle and syringe

Kimwipe "A" was used to absorb undelivered fluid present on the surface of the skin for 30 seconds post-injection.

Kimwipe "B" was used to absorb any fluid present on the surface of the skin 5 minutes post-injection.

Kimwipes "A" and "B" were not contaminated with blood from injection, unless otherwise noted.

**Table S2: Summary of Delivered Dose Determination Administration With Needle-Free Injector**

| Group | | 2 | | | |
| --- | --- | --- | --- | --- | --- |
| Animal Number | | 1 | 2 | 3 | 4 |
| Device Name | | 2 mL Prototype needle-free injector | | | |
| Serial Number | | CUSP-04 | | | |
| Route of Administration | | Subcutaneous | | | |
| Location of Treatment | | Left Lower Abdomen | Right Lower Abdomen | Right Lower Abdomen | Left Lower Abdomen |
| Pre- Injection | Weight of Cartridge with Tip (g) | 5.7006 | 5.6975 | 5.7243 | 5.7081 |
|  | Weight of Kimwipe "A" (g) | 0.4708 | 0.4645 | 0.4676 | 0.4460 |
|  | Weight of Kimwipe "B" (g) | 0.4650 | 0.4693 | 0.4473 | 0.4418 |
|  | Weight of Kimwipe "C" (g) | 0.4589 | 0.4637 | 0.4491 | 0.4468 |
| Post- Injection | Weight of Cartridge with Tip (g) | 3.4617 | 3.4630 | 3.4577 | 3.4600 |
|  | Weight of Kimwipe "A" (g) | 0.4957 | 0.4954 | 0.5836 | 0.4665 |
|  | Weight of Kimwipe "B" (g) | 0.4950 | 0.5377 | 0.4694 | 0.4856 |
|  | Weight of Kimwipe "C" (g) | 0.4596 | 0.5642 | 0.5200 | 0.4469 |
| Weight of Fluid Delivered with Cartridge with Tip (g) | | 2.2389 | 2.2345 | 2.2666 | 2.2481 |
| Weight of Fluid Absorbed in Kimwipe "A" (g) | | 0.0249 | 0.0309 | 0.1160 | 0.0205 |
| Weight of Fluid Absorbed in Kimwipe "B" (g) | | 0.0300 | 0.0684 | 0.0221 | 0.0438 |
| Weight of Fluid Absorbed in Kimwipe "C" (g) | | 0.0007 | 0.1005 | 0.0709 | 0.0001 |
| Total Weight of Undelivered Fluid (g) | | 0.0556 | 0.1998 | 0.2090 | 0.0644 |
| Procedural Comments | | None | Kimwipes “A” and “C” contaminated with blood from injection. | Kimwipes “A”, “B”, and “C” contaminated with blood from injection. | None |

Kimwipe "A" was used to absorb undelivered fluid present on the surface of the skin for 30 seconds post-injection.

Kimwipe "B" was used to wipe the front end of the device to absorb the fluid present on the device.

Kimwipe "C" was used to absorb any fluid present on the surface of the skin 5 minutes post-injection.

Kimwipes "A", "B", and "C" were not contaminated with blood from injection, unless otherwise noted.

**Table S3: Body Condition Score Matrix**

| Large Animal Body Condition Score Descriptions | | |
| --- | --- | --- |
| Score | General Appearance | Structural Descriptor |
| 1.0 | Emaciated | Bone structure readily apparent; Ribs, hips and backbone visible; Bony protuberances easily palpated with no fat coverage. |
| 1.5 | Very thin | Ribs, hips and spine visible. |
| 2.0 | Thin | Ribs not visible; Hips visible and spine only visible near scapulae; Ribs, hips and backbone noticeable and easily palpated. |
| 2.5 | Lean but Good Muscle Tone | Tubular shaped but waist and flatness at sides evident; Ribs, hips and backbone palpated with minimal pressure. |
| 3.0 | Normal | Tubular shape with slight rounding of the sides; Ribs, hips and backbone palpated with pressure. |
| 3.5 | Good Condition | Tubular shape; Ribs, hips and backbone palpated with firm pressure. |
| 4.0 | Well-Covered | Slight bulbous shape with noticeable fat accumulations; Ribs, hips and backbone palpated with difficulty. |
| 4.5 | Well-Covered with Apparent Fat Accumulations | Bulbous shape; Ribs, hips and backbone not palpable. |
| 5.0 | Overfat/Obese | Rectangular shape is generalized; Ribs, hips and backbone heavily covered and not palpable. |

**Table S4: Animal Body Condition**

| Group | Animal Number | Score Day 0 | Week 1 | Week 2 | Week 3 | Week 4 | Week 5 | Week 6 | Week 7 | Week 8 | Week 9 | Body Condition Score at Euthanasia |
| --- | --- | --- | --- | --- | --- | --- | --- | --- | --- | --- | --- | --- |
| 1 | 1 | 3.0 | 3.0 | 3.0 | 3.0 | 3.0 | 3.0 | 3.0 | 3.0 | 3.0 | 3.0 | 3.0 |
|  | 2 | 3.0 | 3.0 | 3.0 | 3.0 | 3.0 | 3.0 | 3.0 | 3.0 | 3.0 | 3.0 | 3.0 |
|  | 3 | 3.0 | 3.0 | 3.0 | 3.0 | 3.0 | 3.0 | 3.0 | 3.0 | 3.0 | 3.0 | 3.0 |
|  | 4 | 3.0 | 3.0 | 3.0 | 3.0 | 3.0 | 3.0 | 3.0 | 3.0 | 3.0 | 3.0 | 3.0 |
| 2 | 1 | 3.0 | 3.0 | 3.0 | 3.0 | 3.0 | 3.0 | 3.0 | 3.0 | 3.0 | 3.0 | 3.0 |
|  | 2 | 3.0 | 3.0 | 3.0 | 3.0 | 3.0 | 3.0 | 3.0 | 3.0 | 3.0 | 3.0 | 3.5 |
|  | 3 | 3.0 | 3.0 | 3.0 | 3.0 | 3.0 | 3.0 | 3.0 | 3.0 | 3.0 | 3.0 | 3.0 |
|  | 4 | 3.0 | 3.0 | 3.0 | 3.0 | 3.0 | 3.0 | 3.0 | 3.0 | 3.0 | 3.0 | 3.0 |
| 3 | 1 | 3.0 | 3.0 | 3.0 | 3.0 | 3.0 | 3.0 | 3.0 | 3.0 | 3.0 | 3.0 | 3.0 |
|  | 2 | 3.0 | 3.0 | 3.0 | 3.0 | 3.0 | 3.0 | 3.0 | 3.0 | 3.0 | 3.0 | 3.0 |
|  | 3 | 3.0 | 3.0 | 3.0 | 3.0 | 3.0 | 3.0 | 3.0 | 3.0 | 3.0 | 3.0 | 3.0 |
|  | 4 | 3.0 | 3.0 | 3.0 | 3.0 | 3.0 | 3.0 | 3.0 | 3.0 | 3.0 | 3.0 | 3.0 |

**Table S5: Animal Body Weights**

| Group | Animal Number | Body Weight on Day 0 (kg) | Week 1 (kg) | Week 2 (kg) | Week 3 (kg) | Week 4 (kg) | Week 5 (kg) | Week 6 (kg) | Week 7 (kg) | Week 8 (kg) | Week 9 (kg) | Body Weight at Euthanasia (kg) | % Body Weight Change |
| --- | --- | --- | --- | --- | --- | --- | --- | --- | --- | --- | --- | --- | --- |
| 1 | 1 | 55.1 | 58.2 | 60.8 | 63.5 | 67.0 | 72.0 | 73.7 | 81.9 | 82.8 | 89.7 | 93.6 | 70% |
|  | 2 | 56.2 | 61.9 | 64.2 | 69.8 | 72.5 | 77.1 | 80.6 | 84.5 | 88.6 | 96.3 | 99.0 | 76% |
|  | 3 | 51.9 | 57.0 | 61.0 | 64.5 | 66.2 | 71.6 | 74.4 | 79.6 | 81.2 | 84.4 | 86.3 | 66% |
|  | 4 | 60.5 | 64.2 | 68.4 | 69.4 | 67.2 | 71.6 | 74.0 | 76.9 | 78.3 | 84.1 | 88.6 | 46% |
| 2 | 1 | 57.5 | 65.7 | 68.1 | 72.4 | 77.3 | 80.7 | 87.3 | 89.7 | 92.6 | 100.2 | 102.4 | 78% |
|  | 2 | 59.0 | 63.7 | 65.8 | 70.6 | 74.3 | 77.5 | 79.8 | 84.5 | 89.7 | 92.3 | 96.0 | 63% |
|  | 3 | 59.9 | 66.5 | 69.1 | 73.4 | 77.7 | 84.5 | 90.4 | 96.3 | 103.2 | 104.3 | 113.0 | 89% |
|  | 4 | 54.0 | 57.9 | 59.5 | 62.3 | 66.1 | 69.4 | 73.2 | 77.9 | 82.7 | 86.8 | 91.6 | 70% |
| 3 | 1 | 60.6 | 64.3 | 67.3 | 71.3 | 71.5 | 75.3 | 79.2 | 81.7 | 88.7 | 89.2 | 93.6 | 54% |
|  | 2 | 58.9 | 64.1 | 65.2 | 74.2 | 75.6 | 81.6 | 87.2 | 93.0 | 99.4 | 104.1 | 109.3 | 86% |
|  | 3 | 55.5 | 60.5 | 62.3 | 68.4 | 73.9 | 75.5 | 78.2 | 81.1 | 89.4 | 92.6 | 93.7 | 69% |
|  | 4 | 49.0 | 53.1 | 56.0 | 63.1 | 66.3 | 69.4 | 73.8 | 78.5 | 83.5 | 91.5 | 92.0 | 88% |

**Table S6: Draize Scoring for Group 1 – Animal 1 (Needle and Syringe)**

| Group | | 1 | | | |
| --- | --- | --- | --- | --- | --- |
| Animal Number | | 1 | | | |
| Treatment Site | | Right Lower Abdomen | | | |
| Treatment Date | | 10/02/23 | | | |
| Study Date | Study Day Post Treatment | Skin Reaction Scoring | | Irritation Score | Site Observation Comments |
|  |  | Erythema | Edema |  |  |
| 02 Oct 2023 | Prior to Injection | 0 | 0 | 0 | None |
|  | Upon Injection Completion | NR | NR |  | NR |
|  | 30 Seconds | 1 | 0 | 1 | None |
|  | 1 Minute | 1 | 0 | 1 |  |
|  | 2 Minutes | 1 | 0 | 1 |  |
|  | 5 Minutes | 0 | 0 | 0 |  |
|  | 10 Minutes | 0 | 0 | 0 |  |
|  | 20 Minutes | 0 | 0 | 0 |  |
|  | 2 Hours | 0 | 0 | 0 |  |
|  | 3 Hours | 0 | 0 | 0 |  |
| 03 Oct 2023 | 1 | 0 | 0 | 0 |  |
| 04 Oct 2023 | 2 | 0 | 0 | 0 |  |
| 05 Oct 2023 | 3 | 0 | 0 | 0 |  |
| 06 Oct 2023 | 4 | 0 | 0 | 0 |  |
| 07 Oct 2023 | 5 | 0 | 0 | 0 |  |

NR = Not recorded

**Table S7: Draize Scoring for Group 1 – Animal 2 (Needle and Syringe)**

| Group | | 1 | | | |
| --- | --- | --- | --- | --- | --- |
| Animal Number | | 2 | | | |
| Treatment Site | | Left Lower Abdomen | | | |
| Treatment Date | | 10/02/23 | | | |
| Study Date | Study Day Post Treatment | Skin Reaction Scoring | | Irritation Score | Site Observation Comments |
|  |  | Erythema | Edema |  |  |
| 02 Oct 2023 | Prior to Injection | 0 | 0 | 0 | None |
|  | Upon Injection Completion | NR | NR |  | NR |
|  | 30 Seconds | 1 | 0 | 1 | None |
|  | 1 Minute | 1 | 0 | 1 |  |
|  | 2 Minutes | 1 | 0 | 1 |  |
|  | 5 Minutes | 1 | 0 | 1 |  |
|  | 10 Minutes | 1 | 0 | 1 | Fluid droplet present at injection site |
|  | 20 Minutes | 1 | 0 | 1 |  |
|  | 2 Hours | 1 | 0 | 1 | None |
|  | 3 Hours | 0 | 0 | 0 |  |
| 03 Oct 2023 | 1 | 0 | 0 | 0 |  |
| 04 Oct 2023 | 2 | 0 | 0 | 0 |  |
| 05 Oct 2023 | 3 | 0 | 0 | 0 |  |
| 06 Oct 2023 | 4 | 0 | 0 | 0 |  |
| 07 Oct 2023 | 5 | 0 | 0 | 0 |  |

NR = Not recorded

**Table S8: Draize Scoring for Group 1 – Animal 3 (Needle and Syringe)**

| Group | | | 1 | | | |
| --- | --- | --- | --- | --- | --- | --- |
| Animal Number | | | 3 | | | |
| Treatment Site | | | Right Lower Abdomen | | | |
| Treatment Date | | | 10/02/23 | | | |
| Study Date | Study Day Post Treatment | Skin Reaction Scoring | | | Irritation Score | Site Observation Comments |
|  |  | Erythema | | Edema |  |  |
| 02 Oct 2023 | Prior to Injection | 1 | | 0 | 1 | None |
|  | Upon Injection Completion | NR | | NR |  | NR |
|  | 30 Seconds | 1 | | 1 | 2 | None |
|  | 1 Minute | 1 | | 1 | 2 |  |
|  | 2 Minutes | 0 | | 1 | 1 |  |
|  | 5 Minutes | 0 | | 1 | 1 |  |
|  | 10 Minutes | 0 | | 1 | 1 | Blood spot at injection site |
|  | 20 Minutes | 0 | | 1 | 1 |  |
|  | 2 Hours | 0 | | 1 | 1 | None |
|  | 3 Hours | 0 | | 0 | 0 |  |
| 03 Oct 2023 | 1 | 0 | | 0 | 0 |  |
| 04 Oct 2023 | 2 | 0 | | 0 | 0 |  |
| 05 Oct 2023 | 3 | 0 | | 0 | 0 |  |
| 06 Oct 2023 | 4 | 0 | | 0 | 0 |  |
| 07 Oct 2023 | 5 | 0 | | 0 | 0 |  |

NR = Not recorded

**Table S9: Draize Scoring for Group 1 – Animal 4 (Needle and Syringe)**

| Group | | 1 | | | |
| --- | --- | --- | --- | --- | --- |
| Animal Number | | 4 | | | |
| Treatment Site | | Left Lower Abdomen | | | |
| Treatment Date | | 10/02/23 | | | |
| Study Date | Study Day Post Treatment | Skin Reaction Scoring | | Irritation Score | Site Observation Comments |
|  |  | Erythema | Edema |  |  |
| 02 Oct 2023 | Prior to Injection | 0 | 0 | 0 | None |
|  | Upon Injection Completion | NR | NR |  | NR |
|  | 30 Seconds | 0 | 1 | 1 | None |
|  | 1 Minute | 0 | 1 | 1 |  |
|  | 2 Minutes | 0 | 1 | 1 | Fluid droplet present at injection site. |
|  | 5 Minutes | 0 | 1 | 1 |  |
|  | 10 Minutes | 0 | 1 | 1 |  |
|  | 20 Minutes | 0 | 1 | 1 | None |
|  | 2 Hours | 0 | 0 | 0 |  |
|  | 3 Hours | 0 | 0 | 0 |  |
| 03 Oct 2023 | 1 | 0 | 0 | 0 |  |
| 04 Oct 2023 | 2 | 0 | 0 | 0 |  |
| 05 Oct 2023 | 3 | 0 | 0 | 0 |  |
| 06 Oct 2023 | 4 | 0 | 0 | 0 |  |
| 07 Oct 2023 | 5 | 0 | 0 | 0 |  |

NR = Not recorded

**Table S10: Draize Scoring for Group 2 – Animal 1 (Needle and Syringe)**

| Group | | 2 | | | |
| --- | --- | --- | --- | --- | --- |
| Animal Number | | 1 | | | |
| Treatment Site | | Left Lower Abdomen | | | |
| Treatment Date | | 09/05/23 | | | |
| Study Date | Study Day Post Treatment | Skin Reaction Scoring | | Irritation Score | Site Observation Comments |
|  |  | Erythema | Edema |  |  |
| 05 Sep 2023 | Prior to Injection | 0 | 0 | 0 | None |
|  | Upon Injection Completion | NR | NR |  | NR |
|  | 30 Seconds | 0 | 1 | 1 | None |
|  | 1 Minute | 0 | 1 | 1 |  |
|  | 2 Minutes | 0 | 1 | 1 |  |
|  | 5 Minutes | 0 | 1 | 1 |  |
|  | 10 Minutes | 0 | 1 | 1 |  |
|  | 20 Minutes | 0 | 1 | 1 |  |
|  | 2 Hours | 0 | 0 | 0 |  |
|  | 3 Hours | 0 | 0 | 0 |  |
| 06 Sep 2023 | 1 | 1 | 0 | 1 |  |
| 07 Sep 2023 | 2 | 0 | 0 | 0 |  |
| 08 Sep 2023 | 3 | 0 | 0 | 0 |  |
| 09 Sep 2023 | 4 | 0 | 0 | 0 |  |
| 10 Sep 2023 | 5 | 0 | 0 | 0 |  |

NR = Not recorded

**Table S11: Draize Scoring for Group 2 – Animal 2 (Needle and Syringe)**

| Group | | 2 | | | |
| --- | --- | --- | --- | --- | --- |
| Animal Number | | 2 | | | |
| Treatment Site | | Right Lower Abdomen | | | |
| Treatment Date | | 09/05/23 | | | |
| Study Date | Study Day Post Treatment | Skin Reaction Scoring | | Irritation Score | Site Observation Comments |
|  |  | Erythema | Edema |  |  |
| 05 Sep 2023 | Prior to Injection | 0 | 0 | 0 | None |
|  | Upon Injection Completion | NR | NR |  | NR |
|  | 30 Seconds | 0 | 1 | 1 | None |
|  | 1 Minute | 0 | 1 | 1 |  |
|  | 2 Minutes | 0 | 1 | 1 |  |
|  | 5 Minutes | 0 | 1 | 1 |  |
|  | 10 Minutes | 0 | 1 | 1 | Some fluid observed at injection site. |
|  | 20 Minutes | 0 | 1 | 1 | None |
|  | 2 Hours | 0 | 0 | 0 |  |
|  | 3 Hours | 0 | 0 | 0 |  |
| 06 Sep 2023 | 1 | 0 | 0 | 0 |  |
| 07 Sep 2023 | 2 | 0 | 0 | 0 |  |
| 08 Sep 2023 | 3 | 0 | 0 | 0 |  |
| 09 Sep 2023 | 4 | 0 | 0 | 0 |  |
| 10 Sep 2023 | 5 | 0 | 0 | 0 |  |

NR = Not recorded

**Table S12: Draize Scoring for Group 2 – Animal 3 (Needle and Syringe)**

| Group | | 2 | | | |
| --- | --- | --- | --- | --- | --- |
| Animal Number | | 23-19-3 | | | |
| Treatment Site | | Right Lower Abdomen | | | |
| Treatment Date | | 10/02/23 | | | |
| Study Date | Study Day Post Treatment | Skin Reaction Scoring | | Irritation Score | Site Observation Comments |
|  |  | Erythema | Edema |  |  |
| 02 Oct 2023 | Prior to Injection | 0 | 0 | 0 | None |
|  | Upon Injection Completion | NR | NR |  | NR |
|  | 30 Seconds | 1 | 1 | 2 | Approximately 0.1 mL of blood seeping from injection site |
|  | 1 Minute | 1 | 1 | 2 |  |
|  | 2 Minutes | 1 | 1 | 2 |  |
|  | 5 Minutes | 1 | 1 | 2 |  |
|  | 10 Minutes | 1 | 1 | 2 | None |
|  | 20 Minutes | 1 | 1 | 2 |  |
|  | 2 Hours | 1 | 1 | 2 |  |
|  | 3 Hours | 0 | 0 | 0 |  |
| 03 Oct 2023 | 1 | 0 | 0 | 0 |  |
| 04 Oct 2023 | 2 | 0 | 0 | 0 |  |
| 05 Oct 2023 | 3 | 0 | 0 | 0 |  |
| 06 Oct 2023 | 4 | 0 | 0 | 0 |  |
| 07 Oct 2023 | 5 | 0 | 0 | 0 |  |

NR = Not recorded

**Table S13: Draize Scoring for Group 2 – Animal 4 (Needle and Syringe)**

| Group | | 2 | | | |
| --- | --- | --- | --- | --- | --- |
| Animal Number | | 23-20-6 | | | |
| Treatment Site | | Left Lower Abdomen | | | |
| Treatment Date | | 10/02/23 | | | |
| Study Date | Study Day Post Treatment | Skin Reaction Scoring | | Irritation Score | Site Observation Comments |
|  |  | Erythema | Edema |  |  |
| 02 Oct 2023 | Prior to Injection | 0 | 0 | 0 | None |
|  | Upon Injection Completion | NR | NR |  | NR |
|  | 30 Seconds | 0 | 1 | 1 | None |
|  | 1 Minute | 0 | 1 | 1 |  |
|  | 2 Minute | 0 | 1 | 1 |  |
|  | 5 Minute | 0 | 1 | 1 |  |
|  | 10 Minute | 0 | 1 | 1 | Some fluid observed at injection site |
|  | 20 Minute | 1 | 1 | 2 | None |
|  | 2 Hour | 0 | 1 | 1 |  |
|  | 3 Hours | 0 | 1 | 1 |  |
| 03 Oct 2023 | 1 | 0 | 0 | 0 |  |
| 04 Oct 2023 | 2 | 0 | 0 | 0 |  |
| 05 Oct 2023 | 3 | 0 | 0 | 0 |  |
| 06 Oct 2023 | 4 | 0 | 0 | 0 |  |
| 07 Oct 2023 | 5 | 0 | 0 | 0 |  |

NR = Not recorded

**Table S14: Mean Concentrations of mAb A in Serum Following Administration**

| Study Day | Time Post Dose (h) | mAb A 2mL SC | | | | mAb A 2mL SC (NFI) | | | | mAb A 2mL IV | | | |
| --- | --- | --- | --- | --- | --- | --- | --- | --- | --- | --- | --- | --- | --- |
|  |  | n | Mean | SD | CV | n | Mean | SD | CV | n | Mean | SD | CV |
|  |  |  | (μg/mL) | (μg/mL) | (%) |  | (μg/mL) | (μg/mL) | (%) |  | (μg/mL) | (μg/mL) | (%) |
| D1 | PREDOSE | 4 | BLQ | NC | NC | 4 | BLQ | NC | NC | 4 | BLQ | NC | NC |
| D1 | 0.25 | 0 | NC | NC | NC | 0 | NC | NC | NC | 4 | 142 | 30.4 | 21.4 |
| D1 | 6 | 4 | 33.8 | 17.0 | 50.2 | 4 | 22.9 | 8.16 | 35.6 | 4 | 127 | 26.7 | 21.0 |
| D1 | 12 | 4 | 45.2 | 24.0 | 53.0 | 4 | 34.4 | 6.64 | 19.3 | 4 | 108 | 21.4 | 19.8 |
| D2 | 24 | 4 | 53.3 | 19.8 | 37.1 | 4 | 46.4 | 5.60 | 12.1 | 4 | 94.4 | 9.96 | 10.6 |
| D3 | 48 | 4 | 59.7 | 19.0 | 31.8 | 4 | 52.8 | 3.29 | 6.24 | 4 | 79.8 | 11.8 | 14.8 |
| D4 | 72 | 4 | 53.9 | 12.8 | 23.7 | 4 | 54.1 | 2.00 | 3.70 | 4 | 67.3 | 9.69 | 14.4 |
| D5 | 96 | 4 | 52.9 | 12.5 | 23.6 | 4 | 54.6 | 3.34 | 6.12 | 4 | 67.6 | 7.24 | 10.7 |
| D6 | 120 | 4 | 50.9 | 12.1 | 23.8 | 4 | 53.9 | 3.60 | 6.68 | 4 | 62.7 | 6.86 | 11.0 |
| D7 | 144 | 4 | 49.7 | 11.5 | 23.2 | 4 | 51.9 | 2.63 | 5.06 | 4 | 59.5 | 7.60 | 12.8 |
| D8 | 168 | 4 | 48.4 | 10.2 | 21.1 | 4 | 48.8 | 4.15 | 8.50 | 4 | 58.8 | 13.1 | 22.2 |
| D15 | 336 | 4 | 37.1 | 8.75 | 23.6 | 4 | 37.8 | 3.25 | 8.61 | 4 | 43.9 | 6.09 | 13.9 |
| D22 | 504 | 4 | 29.3 | 9.98 | 34.1 | 4 | 32.5 | 6.52 | 20.0 | 4 | 32.8 | 4.04 | 12.3 |
| D29 | 672 | 4 | 21.3 | 8.35 | 39.3 | 4 | 23.9 | 2.95 | 12.3 | 4 | 26.0 | 3.74 | 14.4 |
| D36 | 840 | 4 | 17.4 | 6.61 | 38.0 | 4 | 15.6 | 2.31 | 14.8 | 4 | 17.5 | 3.79 | 21.7 |
| D43 | 1008 | 4 | 12.9 | 5.41 | 41.9 | 4 | 11.8 | 1.61 | 13.6 | 4 | 15.4 | 3.30 | 21.5 |
| D50 | 1176 | 4 | 9.78 | 4.47 | 45.7 | 4 | 9.56 | 1.73 | 18.1 | 4 | 11.5 | 1.52 | 13.3 |
| D57 | 1344 | 4 | 7.61 | 3.78 | 49.8 | 4 | 6.92 | 1.71 | 24.7 | 4 | 9.72 | 1.76 | 18.1 |
| D64 | 1512 | 4 | 5.71 | 2.85 | 49.9 | 4 | 5.29 | 1.32 | 25.1 | 4 | 6.29 | 1.16 | 18.5 |
| D71 | 1680 | 4 | 4.17 | 2.07 | 49.7 | 4 | 3.91 | 0.965 | 24.7 | 4 | 5.71 | 0.655 | 11.5 |

BLQ, Below the limit of quantification; CV, Coefficient of variation; IV, Intravenous; mAb, Monoclonal antibody; n, Number of animals; NC, Not calculated; NFI, Prototype needle-free injector; SC, Subcutaneous; SD, Standard deviation

**Table S15: Pharmacokinetic Parameters for Individual Animals Administered mAb A with Subcutaneous Needle and Syringe**

| Parameter | Unit | mAb A 2mL SC | | | |
| --- | --- | --- | --- | --- | --- |
|  |  | SC#1 F | SC#2 F | SC#3 F | SC#4 F |
| C_max_ | μg/mL | 35.7 | 73.2 | 58.5 | 81.9 |
| T_max_ | day | 2.00 | 0.500 | 2.00 | 2.00 |
| AUC_last_ | day (μg/mL) | 844 | 1650 | 1670 | 1950 |
| AUC_inf_ | day (μg/mL) | 876 | 1730 | 1830 | 2070 |
| t_1/2_ | day | 14.5 | 15.8 | 19.7 | 14.3 |
| CL/F | mL/day | 371 | 188 | 178 | 157 |
| AUC_inf_ % Extrapolated | % | 3.67 | 4.62 | 8.65 | 6.04 |

AUC, Area under the concentration-time curve; AUC_inf_, AUC from time zero extrapolated to infinity; AUC_last_, AUC at the last recorded time point; C_max_, Peak concentration; CL/F, Apparent clearance for SC dosing; F, Female; mAb, Monoclonal antibody; SC, Subcutaneous; t_1/2_, Half-life; T_max_, Time to C_max_

**Table S16: Pharmacokinetic Parameters for Individual Animals Administered mAb A with Subcutaneous NFI**

| Parameter | Unit | mAb A 2mL SC NFI | | | |
| --- | --- | --- | --- | --- | --- |
|  |  | NFI #1 F | NFI #2 F | NFI #3 F | NFI #4 F |
| C_max_ | μg/mL | 58.2 | 58.8 | 54.9 | 52.3 |
| T_max_ | day | 4.00 | 5.00 | 3.00 | 4.00 |
| AUC_last_ | day (μg/mL) | 1690 | 1450 | 1480 | 1510 |
| AUC_inf_ | day (μg/mL) | 1780 | 1520 | 1600 | 1650 |
| t_1/2_ | day | 16.4 | 17.0 | 18.8 | 19.6 |
| CL/F | mL/day | 183 | 214 | 203 | 197 |
| AUC_inf_ % Extrapolated | % | 4.82 | 4.35 | 7.50 | 8.35 |

AUC, Area under the concentration-time curve; AUC_inf_, AUC from time zero extrapolated to infinity; AUC_last_, AUC at the last recorded time point; C_max_, Peak concentration; CL/F, Apparent clearance for SC dosing; F, Female; mAb, Monoclonal antibody; NFI, Needle-free injector; NFI, Prototype needle-free injector; SC, Subcutaneous; t_1/2_, Half-life; T_max_, Time to C_max_

**Table S17: Pharmacokinetic Parameters for Individual Animals Administered mAb A through Intravenous Injection**

| Parameter | Unit | mAb A 2mL IV | | | |
| --- | --- | --- | --- | --- | --- |
|  |  | IV #1 F | IV #2 F | IV #3 F | IV #4 F |
| C_max_ | μg/mL | 129 | 174 | 106 | 159 |
| T_max_ | hour | 0.250 | 0.250 | 0.250 | 0.250 |
| AUC_last_ | day (μg/mL) | 1680 | 1860 | 1860 | 2180 |
| AUC_inf_ | day (μg/mL) | 1800 | 2020 | 2010 | 2360 |
| t_1/2_ | day | 17.8 | 17.6 | 19.2 | 20.9 |
| CL | mL/day | 181 | 161 | 162 | 138 |
| V_ss_ | mL | 4540 | 4050 | 4390 | 3610 |
| AUC_inf_ % Extrapolated | % | 6.85 | 7.86 | 7.82 | 7.75 |

AUC, Area under the concentration-time curve; AUC_inf_, AUC from time zero extrapolated to infinity; AUC_last_, AUC at the last recorded time point; C_max_, Peak concentration; CL, Clearance for IV dosing; F, Female; IV, Intravenous; mAb, Monoclonal antibody; SC, Subcutaneous; t_1/2_, Half-life; T_max_, Time to C_max_; V_ss_, Volume of distribution at steady state

**Table S18: mAb A Serum Concentrations for Individual Animals Dosed with Subcutaneous N&S**

| mAb A 2mL SC | | | | | |
| --- | --- | --- | --- | --- | --- |
| Study Day | Time Post Dose (h) | N&S #1 F | N&S #2 F | N&S #3 F | N&S #4 F |
|  |  | (µg/mL) | | | |
| D1 | PREDOSE | BLQ | BLQ | BLQ | BLQ |
| D1 | 6 | 12.4 | 53.1 | 38.7 | 30.9 |
| D1 | 12 | 15.0 | 73.2 | 49.7 | 43.0 |
| D2 | 24 | 25.8 | 72.9 | 57.1 | 57.3 |
| D3 | 48 | 35.7 | 62.7 | 58.5 | 81.9 |
| D4 | 72 | 35.1 | 62.3 | 56.5 | 61.7 |
| D5 | 96 | 34.2 | 60.0 | 57.7 | 59.6 |
| D6 | 120 | 33.6 | 57.7 | 51.6 | 60.5 |
| D7 | 144 | 33.8 | 60.0 | 48.9 | 55.9 |
| D8 | 168 | 33.3 | 55.5 | 51.2 | 53.4 |
| D15 | 336 | 24.3 | 40.6 | 39.3 | 44.1 |
| D22 | 504 | 15.0 | 31.3 | 32.4 | 38.3 |
| D29 | 672 | 9.47 | 22.6 | 23.9 | 29.1 |
| D36 | 840 | 8.58 | 18.0 | 18.5 | 24.6 |
| D43 | 1008 | 5.76 | 12.2 | 15.1 | 18.5 |
| D50 | 1176 | 3.91 | 8.82 | 12.4 | 14.0 |
| D57 | 1344 | 3.06 | 6.35 | 9.12 | 11.9 |
| D64 | 1512 | 2.09 | 5.06 | 6.90 | 8.78 |
| D71 | 1680 | 1.54 | 3.50 | 5.57 | 6.05 |

BLQ, Below the limit of quantification; F, Female; mAb, Monoclonal antibody; N&S, Needle and syringe; SC, Subcutaneous

**Table S19: mAb A Serum Concentrations for Individual Animals Dosed with Subcutaneous NFI**

| mAb A 2mL SC NFI | | | | | |
| --- | --- | --- | --- | --- | --- |
| Study Day | Time Post Dose (h) | NFI #1 F | NFI #2 F | NFI #3 F | NFI #4 F |
|  |  | (µg/mL) | | | |
| D1 | PREDOSE | BLQ | BLQ | BLQ | BLQ |
| D1 | 6 | 15.1 | 30.6 | 29.3 | 16.7 |
| D1 | 12 | 30.7 | 34.0 | 43.9 | 29.1 |
| D2 | 24 | 53.7 | 45.3 | 46.6 | 40.1 |
| D3 | 48 | 56.3 | 54.4 | 51.5 | 48.8 |
| D4 | 72 | 53.2 | 56.5 | 54.9 | 51.9 |
| D5 | 96 | 58.2 | 56.5 | 51.2 | 52.3 |
| D6 | 120 | 54.3 | 58.8 | 51.0 | 51.4 |
| D7 | 144 | 53.3 | 54.6 | 48.7 | 50.8 |
| D8 | 168 | 54.9 | 47.8 | 45.6 | 47.0 |
| D15 | 336 | 41.4 | 39.4 | 34.1 | 36.2 |
| D22 | 504 | 42.2 | 30.6 | 28.8 | 28.5 |
| D29 | 672 | 28.2 | 23.0 | 23.1 | 21.4 |
| D36 | 840 | 16.1 | 12.7 | 15.3 | 18.3 |
| D43 | 1008 | 11.8 | 9.58 | 12.9 | 13.1 |
| D50 | 1176 | 9.56 | 7.16 | 10.3 | 11.2 |
| D57 | 1344 | 6.64 | 4.76 | 7.42 | 8.87 |
| D64 | 1512 | 5.07 | 3.51 | 6.10 | 6.47 |
| D71 | 1680 | 3.62 | 2.69 | 4.42 | 4.89 |

BLQ, Below the limit of quantification; F, Female; mAb, Monoclonal antibody; NFI, Needle-free injector; NFI, needle-free injector; SC, Subcutaneous

**Table S20: mAb A Serum Concentrations for Individual Animals Dosed with Intravenous Injection**

| mAb A 2mL IV | | | | | |
| --- | --- | --- | --- | --- | --- |
| Study Day | Time Post Dose (h) | IV #1 F | IV #2 F | IV #3 F | IV #4 F |
|  |  | (µg/mL) | | | |
| D1 | PREDOSE | BLQ | BLQ | BLQ | BLQ |
| D1 | 0.25 | 129 | 174 | 106 | 159 |
| D1 | 6 | 115 | 157 | 96.7 | 140 |
| D1 | 12 | 97.7 | 118 | 83.8 | 132 |
| D2 | 24 | 86.7 | 90.8 | 90.9 | 109 |
| D3 | 48 | 70.3 | 90.7 | 68.8 | 89.3 |
| D4 | 72 | 63.9 | 55.3 | 73.3 | 76.8 |
| D5 | 96 | 60.5 | 62.8 | 71.0 | 76.1 |
| D6 | 120 | 56.1 | 57.8 | 66.3 | 70.5 |
| D7 | 144 | 50.7 | 55.9 | 64.0 | 67.4 |
| D8 | 168 | 51.5 | 73.1 | 44.6 | 66.0 |
| D15 | 336 | 36.5 | 44.1 | 43.4 | 51.4 |
| D22 | 504 | 31.9 | 28.2 | 33.0 | 38.0 |
| D29 | 672 | 21.6 | 25.3 | 26.3 | 30.7 |
| D36 | 840 | 17.4 | 12.3 | 19.0 | 21.2 |
| D43 | 1008 | 12.8 | 12.6 | 16.5 | 19.5 |
| D50 | 1176 | 10.4 | 10.2 | 13.5 | 11.7 |
| D57 | 1344 | 7.58 | 11.9 | 9.72 | 9.68 |
| D64 | 1512 | 5.50 | 5.15 | 6.85 | 7.64 |
| D71 | 1680 | 4.79 | 6.26 | 5.69 | 6.08 |

BLQ, Below the limit of quantification; F, Female; IV, Intravenous; mAb, Monoclonal antibody


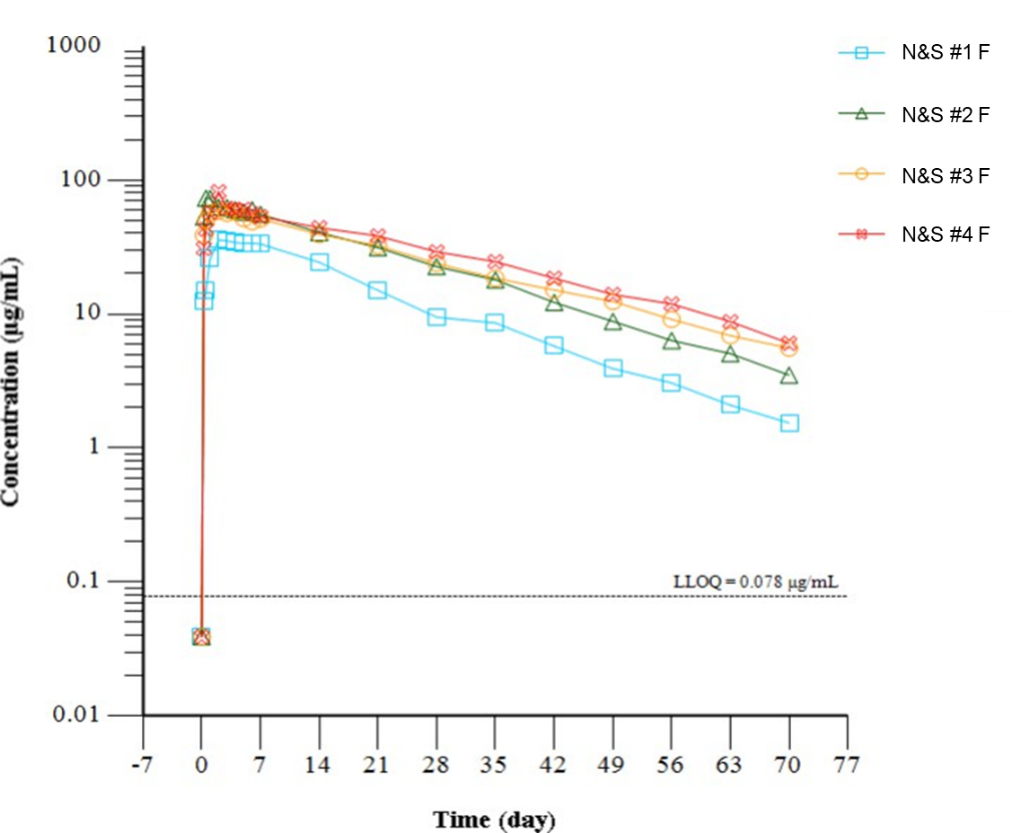


**Figure S2:** Individual concentration vs time profiles for animals administered mAb A through subcutaneous needle and syringe

F, Female; LLOQ, Lower limit of quantitation; mAb, Monoclonal antibody; N&S, Needle and syringe


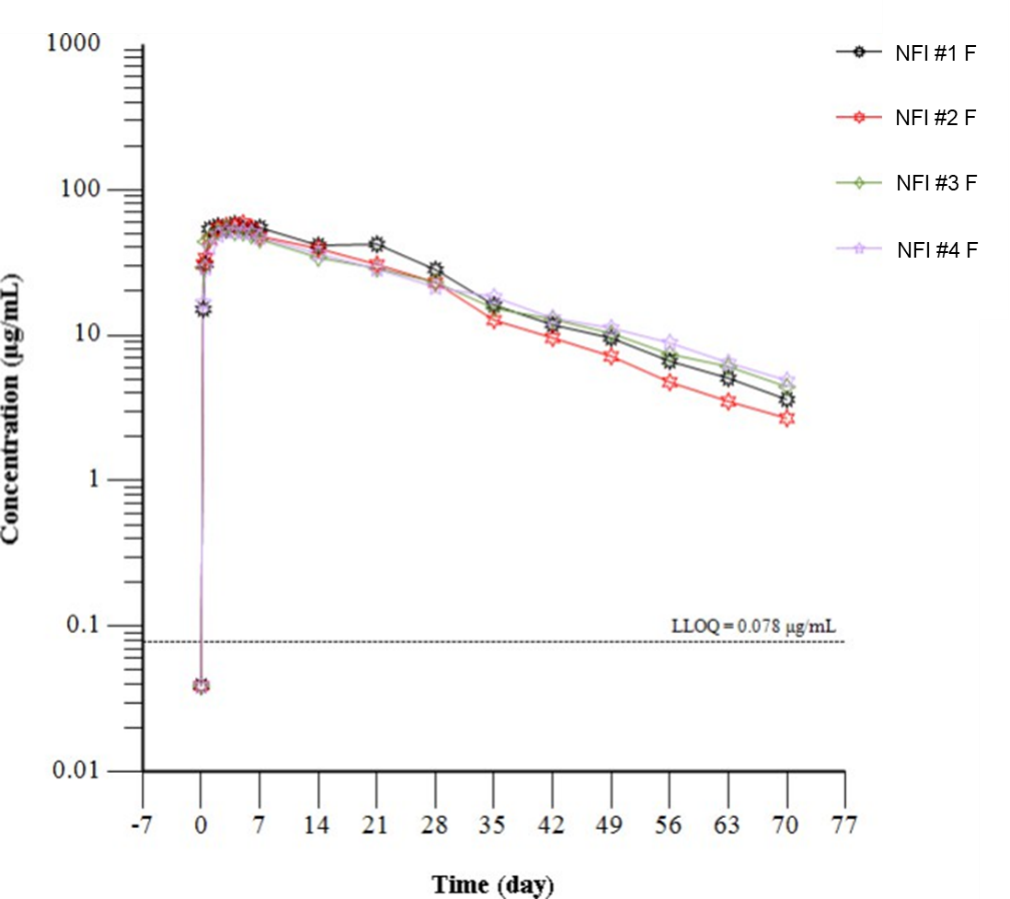


**Figure S3:** Individual concentration vs time profiles for animals administered mAb A through subcutaneous needle-free injection

F, Female; LLOQ, Lower limit of quantitation; mAb, Monoclonal antibody; NFI, Needle-free injector


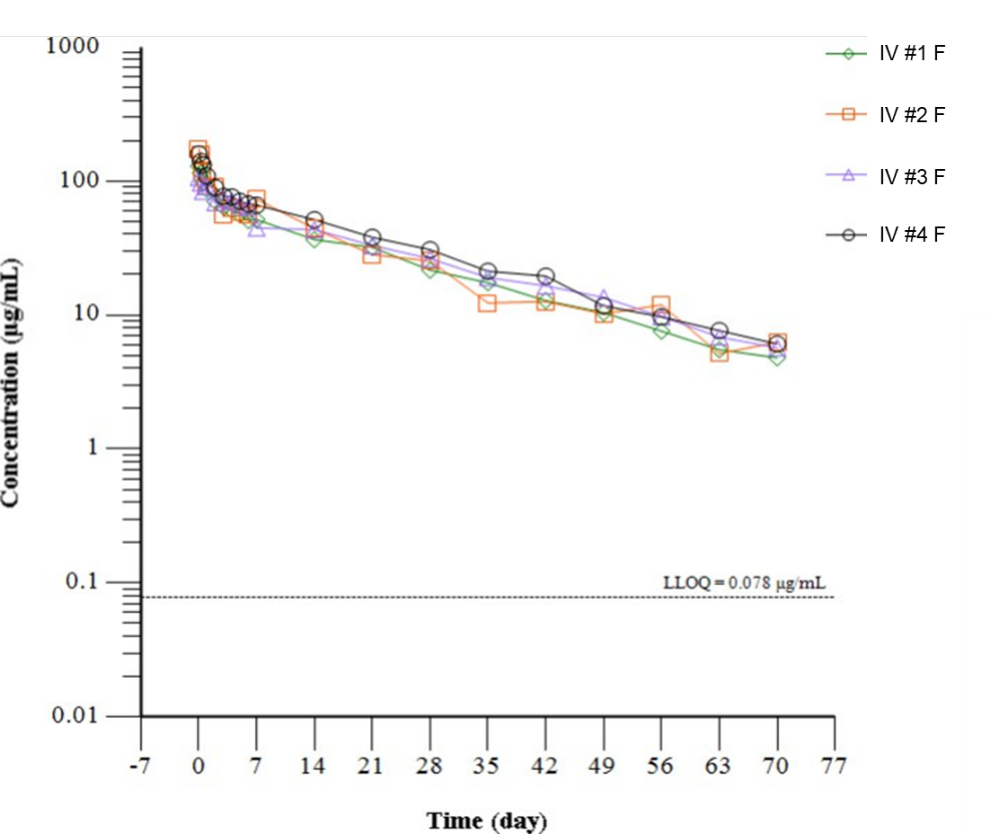


**Figure S4:** Individual concentration vs time profiles for animals administered mAb A through intravenous injection

F, Female; IV, Intravenous; LLOQ, Lower limit of quantitation; mAb, Monoclonal antibody
